# Precision information extraction for rare disease epidemiology at scale

**DOI:** 10.1186/s12967-023-04011-y

**Published:** 2023-02-28

**Authors:** William Z. Kariampuzha, Gioconda Alyea, Sue Qu, Jaleal Sanjak, Ewy Mathé, Eric Sid, Haley Chatelaine, Arjun Yadaw, Yanji Xu, Qian Zhu

**Affiliations:** 1 Division of Rare Diseases Research Innovation, National Center for Advancing Translational Sciences (NCATS), National Institutes of Health (NIH), Bethesda, MD USA; 2 Division of Pre-Clinical Innovation, National Center for Advancing Translational Sciences (NCATS), National Institutes of Health (NIH), 9800 Medical Center Drive, Rockville, MD 20850 USA

## Abstract

**Background:**

The United Nations recently made a call to address the challenges of an estimated 300 million persons worldwide living with a rare disease through the collection, analysis, and dissemination of disaggregated data. Epidemiologic Information (EI) regarding prevalence and incidence data of rare diseases is sparse and current paradigms of identifying, extracting, and curating EI rely upon time-intensive, error-prone manual processes. With these limitations, a clear understanding of the variation in epidemiology and outcomes for rare disease patients is hampered. This challenges the public health of rare diseases patients through a lack of information necessary to prioritize research, policy decisions, therapeutic development, and health system allocations.

**Methods:**

In this study, we developed a newly curated epidemiology corpus for Named Entity Recognition (NER), a deep learning framework, and a novel rare disease epidemiologic information pipeline named EpiPipeline4RD consisting of a web interface and Restful API. For the corpus creation, we programmatically gathered a representative sample of rare disease epidemiologic abstracts, utilized weakly-supervised machine learning techniques to label the dataset, and manually validated the labeled dataset. For the deep learning framework development, we fine-tuned our dataset and adapted the BioBERT model for NER. We measured the performance of our BioBERT model for epidemiology entity recognition quantitatively with precision, recall, and F1 and qualitatively through a comparison with Orphanet. We demonstrated the ability for our pipeline to gather, identify, and extract epidemiology information from rare disease abstracts through three case studies.

**Results:**

We developed a deep learning model to extract EI with overall F1 scores of 0.817 and 0.878, evaluated at the entity-level and token-level respectively, and which achieved comparable qualitative results to Orphanet’s collection paradigm. Additionally, case studies of the rare diseases Classic homocystinuria, GRACILE syndrome, Phenylketonuria demonstrated the adequate recall of abstracts with epidemiology information, high precision of epidemiology information extraction through our deep learning model, and the increased efficiency of EpiPipeline4RD compared to a manual curation paradigm.

**Conclusions:**

EpiPipeline4RD demonstrated high performance of EI extraction from rare disease literature to augment manual curation processes. This automated information curation paradigm will not only effectively empower development of the NIH Genetic and Rare Diseases Information Center (GARD), but also support the public health of the rare disease community.

**Supplementary Information:**

The online version contains supplementary material available at 10.1186/s12967-023-04011-y.

## Introduction

In the United States of America, a rare disease is defined as one that affects fewer than 200,000 people [1]. The European Union defines a rare disease as one that afflicts less than or equal to 5 per 10,000 persons or one which is life-threatening, seriously debilitating, or chronic [2]. Though 84.5% of rare diseases have a prevalence of < 1/1,000,000, the collection of an estimated 7,000 rare diseases[3] is estimated to affect 263–446 million people globally, or 3.5–5.9% of all humans [4]. Rare disease patients face numerous challenges negatively impacting their quality of life, such as a scarcity of accessible health information [5], a small disease-specific community, and a lack of available treatments due to economic limitations in the private sector [6]. To mitigate these challenges, policy makers, funding agencies, and the pharmaceutical industry require information about the epidemiology of a rare disease to estimate the number of patients potentially benefiting from therapeutic development, research funding, and clinical trials [7]. Healthcare systems need such knowledge to address the specific needs of rare disease patients, families, and caregivers. With better understanding of rare disease population burdens, strategies from public health such as screening and prevention could be better implemented [8].

For many common diseases, epidemiology information is collected through regional [9, 10] or national surveys [11–17]. The economies of scale and statistical significance associated with these methods allow for simplified collection, aggregation, and analysis. In contrast, due to their rarity and wide range of prevalence rates, epidemiologic information (EI) on rare diseases must be amalgamated from case reports, epidemiologic studies (ES), and expert opinions [18]. Thus, the methods used to estimate those metrics and the reporting of the metrics, vary significantly. For instance, rare diseases with higher incidence and prevalence, such as cystic fibrosis which affects more than 30,000 people in the United States as of February 2022, can be estimated through the Recommended Uniform Screening Panel [19] and a national patient registry [20]. Syndromes with features that overlap with other diseases usually must be verified from genetic or epigenetic investigations on individual patients suspected of the disease [21]. Thus their incidence and prevalence, such as in the case of Wolf–Hirschhorn syndrome with an incidence rate between 1/20,000 and 1/50,000 in 2008, can only be extrapolated from a small sample [22]. Diseases which are overrepresented in specific subpopulations, such as Hansen’s disease with prevalence of 11.7:10,000 in the Marshallese population in Arkansas between 2003 and 2017, can be estimated from surveillance reports submitted to their local health department [23]. Others with extraordinarily sparse populations, such as acute flaccid myelitis which had an annual incidence of 30 cases in the United States for 2021, are counted when suspected patients are reported to the Centers for Disease Control and Prevention (CDC) and verified [24]. The variety of methods utilized to gather incidence and prevalence of rare diseases increases the complexity of accessing, recognizing, and analyzing the data [25]. Consequently, the data is often incomplete [26], which hinders the standardization of reporting and ease of compilation of EI in a centrally accessible database. Furthermore, continually updating this information manually for a staggering number of rare diseases requires a complex system and significant resources for an organization.

On December 16, 2021, the United Nations adopted a resolution to address “the challenges of persons living with rare diseases and their families.” Specifically expressing concern at the lack of granular data available to nations, they encouraged all member states to “collect, analyze and disseminate disaggregated data on persons living with a rare disease” “which would help identify and address the barriers faced in exercising their human rights.”[27] We aim to act upon this resolution through the efficient and sustainable curation and dissemination of epidemiology data for rare disease patients. The Genetic and Rare Disease Information Center (GARD) [28] managed by the National Center for Advancing Translational Sciences (NIH/NCATS) in the United States aims to compile and curate this information for over 10,000 rare diseases. Currently, GARD curators manually identify and review rare disease related ES from PubMed and genetic and rare diseases databases such as Orphanet [29] and OMIM [30], extract relevant EI from those studies to update GARD, which is tedious and difficult to maintain at scale. Orphanet, whose 41 member countries include much of the European Union as well as Canada, Kazakhstan, and Russia [29], also aims to compile and curate EI for rare diseases and currently follows a similar labor-intensive procedure at a large-scale [18]. The objective of this study is to design and implement Natural Language Processing (NLP) algorithms to identify and extract EI programmatically from rare disease related PubMed articles. We aim for this system to not only aid in internal research efforts and rare disease curation, but also serve as a resource to the public.

Early attempts to extract EI from observational studies utilized rule-based approaches [31]. To ascertain new EI without direct measurements, DisMod II, a tool which calculates a number of different epidemiologic values given a disease’s prevalence and/or incidence, was created [26, 32]. With the digitization of healthcare, electronic medical records have been utilized to estimate epidemiologic rates. However, this has not been easily possible in this domain, as only a limited number of rare diseases are accurately represented with currently existing International Classification of Diseases codes [33, 34]. Automated approaches to clinical epidemiology include “Data extraction for epidemiological research” or DExtER [35]. NLP approaches to information extraction include analyzing social media to analyze drug abuse epidemiology[36] and detecting cancer cases to calculate epidemiologic prevalence [37, 38].

Recently, multiple deep learning approaches to NLP have used Bidirectional Encoder Representations from Transformers[39] (BERT)[40] with self-supervised pre-training on PubMed and PubMed Central and fine-tuned them in a fully-supervised manner to achieve state-of-the-art performance on several biomedical named entity recognition (NER) [41, 42] and entity normalization tasks [43]. In the related clinical domain, pre-training on clinical notes [44–46] and fine-tuning on electronic health records [47] have been demonstrated to identify semantically similar sentences for note summarization [48], classify relations between bleeding events and clinical entities for better detection of bleeding [49], perform clinical entity normalization [47], and predict diseases [50]. Based on the bidirectional transformer’s ability to transfer deep contextual learning and its recent success on a wide variety of NLP tasks, we hypothesize that a BioBERT-based model will be effective for EI extraction [41], particularly given that rare diseases have less training data available. Training this deep learning model for NER requires a dataset labeled at the token level. From querying PubMed and Google Scholar, no datasets labeled with any EI for NER exist. Thus, we believe that weakly-supervised machine learning techniques [51] coupled with manual validation will allow us to create a task-dynamic, high quality dataset for EI extraction with high efficiency. Weakly supervised machine learning [52] encompasses a broad set of techniques such as distant supervision or labeling from existing knowledge sources, prescriptive supervision or labeling using heuristic rules, and noisy supervision or labeling using existing NER models such as spaCy [53]. These approaches have recently become a popular method for achieving analogous results on tasks where the creation of a fully supervised training dataset is not feasible [54]. Our hybrid approach balances the need for high quality annotations in this first-of-a-kind dataset with the labor intensive nature of labeling a dataset from scratch.

Here, we present the first dataset with labeled EI intended for a variety of NLP tasks. To our knowledge, this work also represents the first attempt of using a deep learning framework to extract EI from rare disease epidemiology publications, as well as epidemiology publications in general.

## Methods

**Fig. 1:**
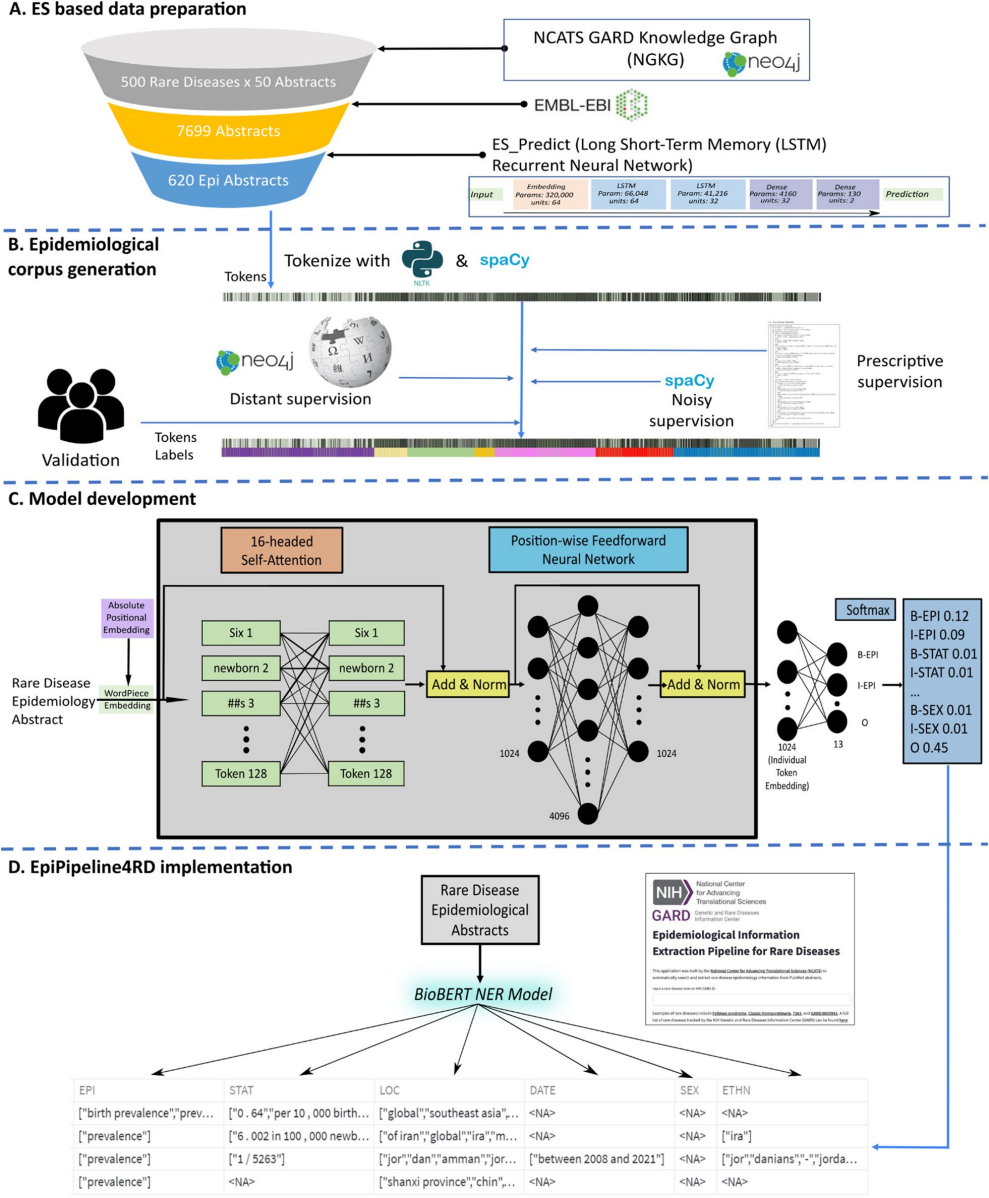
Implementation workflow of EpiPipeline4RD. **A** Steps applied to prepare ES data for deep learning model training. EMBL-EBI refers to the EBI API for gathering abstracts. ES_Predict is a Long Short-Term Memory Recurrent Neural Network for ES prediction. **B** Methods applied for the epidemiology corpus generation. Distant supervision draws upon the NGKG from Neo4J and Wikipedia. Noisy supervision draws upon a spaCy NER model. Prescriptive supervision is dependent upon rules described in the Additional file 2. **C** Transformer model architecture. Positional embeddings are added to the WordPiece embeddings. “Add” refers to the addition of the sub-layer output to its input (residual connection). “Norm” refers to sub-layer normalization after employing a residual connection [55]. **D** EpiPipeline4RD implementation. Output of the EI extraction via the User Interface

To construct an integrated pipeline to extract EI from rare disease ES, we performed four steps sequentially, which are depicted as A to D in the Fig. 1.

### Dataset preparation

We considered the fine-grained EI extraction task as a multi-type token classification or NER task. Training a machine learning model for this task requires a corpus with labeled rare disease epidemiologic information as training data. As no such dataset exists, we created the EI labeled corpus, which is publicly accessible via GitHub (https://github.com/ncats/epi4GARD/tree/master/epi_extract_datasets) and Hugging Face (https://huggingface.co/datasets/ncats/EpiSet4NER-v2).

#### Data retrieval

We randomly selected 500 rare diseases and their synonyms from the NCATS GARD Knowledge Graph (NGKG) [56], which is an integrative knowledge graph containing data from GARD and various biomedical resources, including Orphanet, OMIM, and then we queried the EBI RESTful API [57] to obtain a maximum of 50 PubMed abstracts for each disease. In our previous work [58], we developed ES_Predict, an epidemiologic study predictor based on a long short-term memory based recurrent neural network [59], to predict if a study is ES. We applied the ES_Predict[58] to identify ES related PubMed abstracts, and excluded those with an epidemiologic probability less than 0.5.

#### Data preprocessing

To ensure no false positive ES moved to the next step, we manually reviewed and excluded any irrelevant ES from the dataset. We then split the dataset into training, validation, and test sets. Fifty abstracts were randomly selected as the test set. The remaining abstracts were split into a training set and a hold-out validation set with an approximate 80:20 ratio.

To prepare the data for labeling, we removed HTML remnants and extraneous punctuations (i.e. *, ^, $) present in the abstracts. Additionally, we removed commas from numbers to avoid mis-tokenization in the next step. Notably, we did not remove stopwords nor standardize spaCy entities, including organizations, times, events, persons, quantities, and times, as BERT-based models applied in this study consider them as contextual information to improve predictions [60]. We split each abstract into sentences using Natural Language Tool-Kit (NLTK) [61] and tokenized each sentence using spaCy [53, 62]. We then corrected errata introduced by the spaCy and NLTK tokenizers to ensure that special characters (e.g. a, b, β) were accurately presented, removed whitespace tokens and corrected those incorrect sentence splits, and re-combined numbers split across tokens (e.g. the number “1 000 000” might be split into 3 tokens).

#### Data labeling

**Fig. 2:**
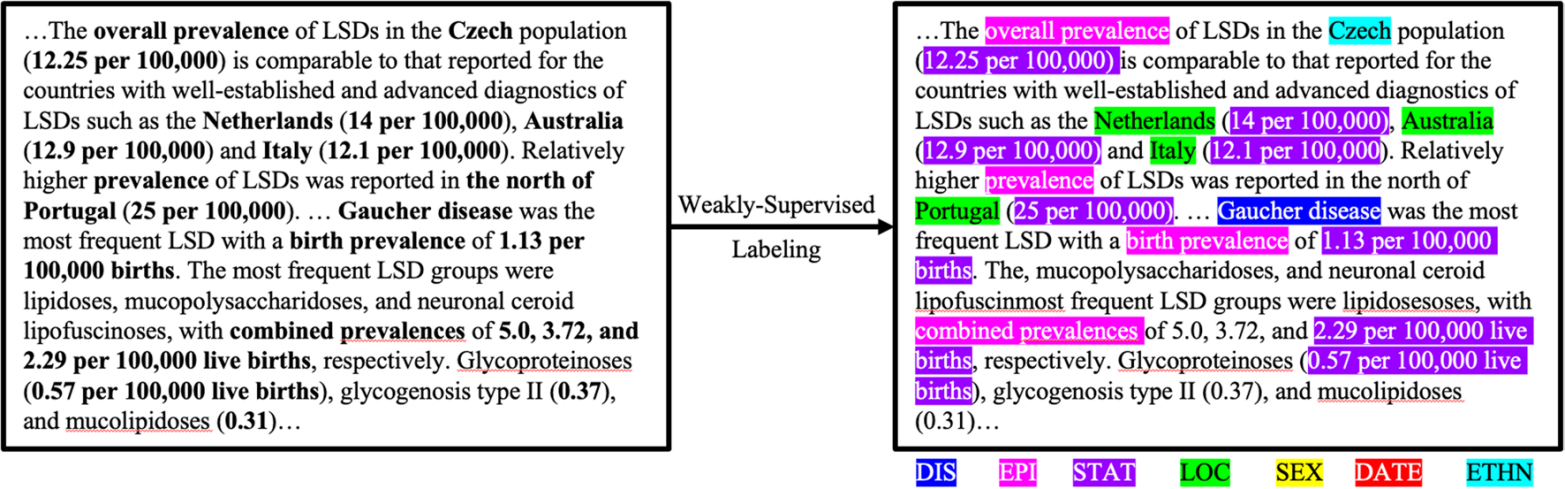
An example of labeling using weakly supervised ML techniques and NLP. Correct labeling is bolded on the left. Actual programmatic output is on the right. Abstract is from [66]

Eight EI relevant entity classes were initially suggested by our subject matter experts (SMEs) (co-authors, GA and ES): epidemiologic type, epidemiologic rate, location, ethnicity/nationality/race, date, sex, disease name and synonym, and disease abbreviation (Table 1). Detailed descriptions of entity classes can be found in Additional file 3. To mitigate a labor-intensive manual labeling process, we developed an algorithm to effectively label the dataset with seven entity classes in the inside-outside-beginning 2 (IOB2) format [63], using NLP and weakly supervised machine learning techniques [51, 52] (Fig. 2). For instance, a location entity of “the United States and Canada” would be split into five individual tokens, “the”, “United”, “States”, “and”, and “Canada” and labeled as “B-LOC”, “I-LOC”, “I-LOC”, “O”, “B-LOC” accordingly, given the definition of IOB2 where “B-(tag)” indicates the beginning of a phrase, “I-(tag)” anything inside the phrase, and the “O” tag indicates anything outside of the phrase.

**Table 1:** Description of eight entity classes in the manually validated dataset

Entity Class	Label	Definition	Example
Disease terms	DIS	Rare and non-rare disease names and synonyms including those which have a unique ID or code (ICD, GARD, UMLS). Includes pathogenic diseases, but not pathogens. Does not include symptoms, features of diseases, phenotypes, nor abbreviations of disease names	“Wegener's granulomatosis”, “Metachromatic leukodystrophy”, “Krabbe disease”
Disease abbreviations	ABRV	Abbreviations of the disease names or synonyms described above	“MPS” (Mucopolysaccharidoses), “FSHD” (Facioscapulohumeral muscular dystrophy)
Epidemiology Type	EPI	The epidemiologic metric being reported	“Annualized incidence”, “point prevalence”, “estimated occurrence rate”
Epidemiology Rate	STAT	The number of people afflicted. Usually expressed as a fraction (rate), a percentage of the (sub)population, or an integer estimation/count of persons with the disease	“Approximately 1 in 40,000 live births”, “50,000 people affected”
Location	LOC	Locations, including geopolitical entities, which indicate where the study took place	“North-Central Africa”, “Salla region of northern Finland”, “the United States”
Dates	DATE	When the study took place or when data was gathered	“Between 1985 and 2006”, “January 21, 1999”
Biological Sex	SEX	Terms that were likely to indicate the biological sex of the persons mentioned in the study	“Men”, “women”, “intersex”
Ethnicity/Nationality/Race	ETHN	Terms that are likely to indicate nationality, race, or ethnicity of the persons afflicted by the disease	“Italian”, “Ashkenazi Jew”, “Marshallese”

Detailed descriptions are listed in Additional file 3

Figure 2 shows an example of weakly supervised labeling. Only four epi rate (STAT) phrases with incomplete forms (missing “per 100,000 live births”) were missed.

#### Manual validation

To ensure the accuracy and quality of the labeled dataset for the BERT model development, we conducted a manual validation by eight biomedical researchers (GA, HC, JS, AY, EM, CQ, YX and QZ) with PhD and MD degrees and one medical school student with BS degree (WK). With the help of our SMEs (GA and ES), we drafted manual validation guidelines (Additional file 3) with detailed descriptions of each entity class and our inclusion/exclusion criteria applied for labeling. We scheduled a training session with all reviewers to assure their processes were consistent with each other and aligned with our requirements. More specifically, we went through the prepared manual validation guidelines and our requirements, which include reviewing each label, correcting any mis-labels, marking any uncertain labels for further review, labeling non-rare diseases as DIS, labeling rare and non-rare disease abbreviations as ABRV and adding any additional notes for further discussion.

Four consecutive validation iterations were performed by four subgroups formed from the aforementioned nine reviewers. For the first pass, we split the entire labeled dataset to three subsets and assigned three co-authors, WK, CQ and QZ, who have first-hand understood of this study and the whole implementation process, to manually validate a subset. For the next round, five co-authors (HC, JS, AY, EM and YX) with various backgrounds ranging from bioinformatics to clinical informatics completed the same process as the first pass on five different subsets. After completing the first two passes, we were confident that most of the mis-labels had been corrected by eight reviewers. However, uncertain labels marked for further review or those with notes from the previous two passes were still unaddressed. One of our SMEs (GA) then took a third pass of the validation process by reviewing and addressing labels marked for review. During this pass, GA flagged any additional mis-labels she observed. In the final pass, WK reviewed all labels with flags from GA and ensured they were labeled optimally for the deep learning model.

### Model development & evaluation

We conducted four steps to develop and evaluate a BioBERT model for EI extraction. (1) We fine-tuned a bidirectional transformer model on our rare disease epidemiology data set. (2) We fine-tuned the dataset by adjusting labels to improve performance. (3) We optimized the model by tuning hyperparameters of the model and evaluating the model on the validation set. (4) We finally tested the model on the test set.

#### Model development

Using the *transformers* Python package [67], we adapted BioBERT large cased v1.1 for NER by concatenating a fully-connected output layer of 13 neural nodes to the end of the transformer encoder because 2*n* + 1 nodes are required in the output layer for *n* entity classes labeled in the IOB2 format. BioBERT large v1.1 is an architecture that produces bidirectional encoder representations from transformers after being pre-trained on English Wikipedia, BooksCorpus, and PubMed abstracts for 1 M steps each. Its tokenizer utilizes the WordPiece algorithm with a vocabulary size of 58,996 [68]. The positional embeddings are absolute. The transformer architecture utilized for this study is illustrated in Fig. 1C. Unless otherwise indicated, the default parameters of BioBERT large v1.1 were utilized.

We then fine-tuned this model using hyperparameters: epochs = 4, learning rate = 5e−5, weight decay = 0.01, maximum sequence length = 128 tokens, training batch size = 16, evaluation batch size = 8, and seed = 42 within the PyTorch framework. Before training, the weight matrices of the nodes were initialized with a standard deviation of 0.02. We tokenized the dataset with a maximum sequence length of 128. The trainer utilized the AdamW stochastic optimization function (β_1_ = 0.9; β_2_ = 0.99; ϵ = 1e−8) [69]. To reduce the probability of overfitting to the small training dataset, each of the hidden layers had a dropout probability of 0.1 [70] and the attention probabilities had a dropout ratio of 0.1 [39].

We initially trained the PyTorch model [71] for 4 epochs with learning rate of 5e−5; weight decay of 0.01; maximum sequence length of 128 tokens; training batch size of 16; seed = 42 on the training set and predicted on the validation set with an evaluation batch size of 8. We calculated precision, recall, and F1 scores at the entity- and token-levels for each individual entity class. Entity-level evaluation considers all tokens in a multi-token entity as a single unit. Thus, if one token in an entity is misclassified, the prediction on the entire entity is marked incorrect. For instance, if one token within a multi-token phrase such as “1 per 50,000 people”, which would be labeled as “B-STAT”, “I-STAT”, “I-STAT”, “I-STAT” is misclassified, the whole entity is marked incorrect. The denominator for precision, recall, and F1 is also the number of entities. We utilized the *seqeval* Python framework to get entity-level metrics [72]. For token-level evaluation, we evaluated each token’s classification independently. For instance, if the phrase “incidence at birth” should be labeled as “B-EPI”, “I-EPI”, and “I-EPI” (See Methods 1C for more details about those labels), but the model incorrectly predicts it as “B-EPI”, “I-EPI”, and “O”, the recall score for those three tokens would be 2/3. Overall token-level evaluation uses micro-averaging [73]. We developed our own algorithm to compare the model’s classification of each individual word to the validated dataset. The algorithm ignored the beginning/inside (“B-”/“I-”) component of the IOB2 tag and calculated precision, recall, and F1 scores for each token in each class.

To assess the performance of BioBERT large cased v1.1 compared to related pretrained models, BioBERT base cased v1.2 [41], PubMedBERT base, PubMedBERT + PMC base [42], as well as BlueBERT base and large [46], which are models pretrained similarly on all of PubMed abstracts. PubMedBERT and BlueBERT models were uncased. We calculated overall F1 score at the entity-level and overall precision at the token-level using micro-averaging for the assessment [73].

#### Dataset finetuning

The complexity and the number of tokens corresponding to eight defined entity classes in the dataset varies significantly among the entity classes, which may negatively impact the overall performance of the model. Thus, we investigated the impact of each entity class on performance of the model of BioBERT large v1.1. It showed that the model had poor performance on DIS, ABRV, and ETHN entities, due to great variation of presentations of disease names and abbreviations as well as limited ethnicity/nationality/racial information available in our training dataset shown in Table 1. We created three variants of the dataset and conducted experiments to identify the optimal dataset variant. They are “Dataset with DIS and ABRV merged”, which was created by converting ABRV labels into DIS labels; “Dataset without ABRV and DIS”, created by replacing disease (DIS) and abbreviation (ABRV) labels with the null label (“O”); and “Dataset without ABRV, DIS, and ETHN”, created by replacing abbreviation (ABRV), disease (DIS), and ethnicity/nationality/race (ETHN) labels with the null label (“O”). BioBERT large v1.1 was then fine-tuned on each dataset variant and then predicted on its respective validation set. The variant dataset with the highest statistical results, i.e., precision, recall and F1 model was chosen for model optimization.

#### Model optimization

In addition, we conducted a few experiments to identify optimal hyperparameters to fine-tune BioBERT large cased v1.1 on our chosen variant dataset. We kept the dataset constant and changed the following hyperparameters: AdamW epsilon (1e−2, 1e−4, 1e−6, 1e−8) [74], weight decay (0.01, 0.05, 0.1), training batch size (16, 32), learning rate (2e−5, 3e−5, 4e−5, 5e−5), warm-up ratio (0.0, 0.05, 0.06) [75], learning rate scheduler (linear, cosine), gradient accumulation steps (1, 2, 4) [76], gradient checkpointing (On, Off) [77, 78], 16-bit mixed precision training (On, Off) [79], training epochs (1,4,5,7). We evaluated each model on the validation set at entity- and token-levels for the entire set as well as the individual entity classes. The model with the highest F1 score was chosen as the final model.

#### Model testing and Orphanet Comparison

The final model was then tested on the test set of 50 abstracts. Precision, recall and F1 score were calculated at entity- and token-levels overall and for the individual entity classes.

To qualitatively assess the validity of the final model for EI extraction, we compared our model’s output to epidemiologic data released by Orphanet [80]. To compare equitably, we excluded Orphanet entries with expert opinions as sources, entries without PubMed IDs, and entries with no listed epi rates (STAT). We limited our comparison to abstracts with mentions of EI, and only compared our extractions that contained at least one epi rate (STAT) and no more than one GARD ID (identified with the disease identification function) to Orphanet’s curated data. To compare epidemiologic rates, we manually normalized the in-text prevalence to per 100,000. We also assigned a location of “Worldwide” to any abstract that did not contain location information to be comparable to Orphanet’s extrapolations [18]. The comparison is presented in the Result section.

### EI extraction pipeline implementation

**Fig. 3:**
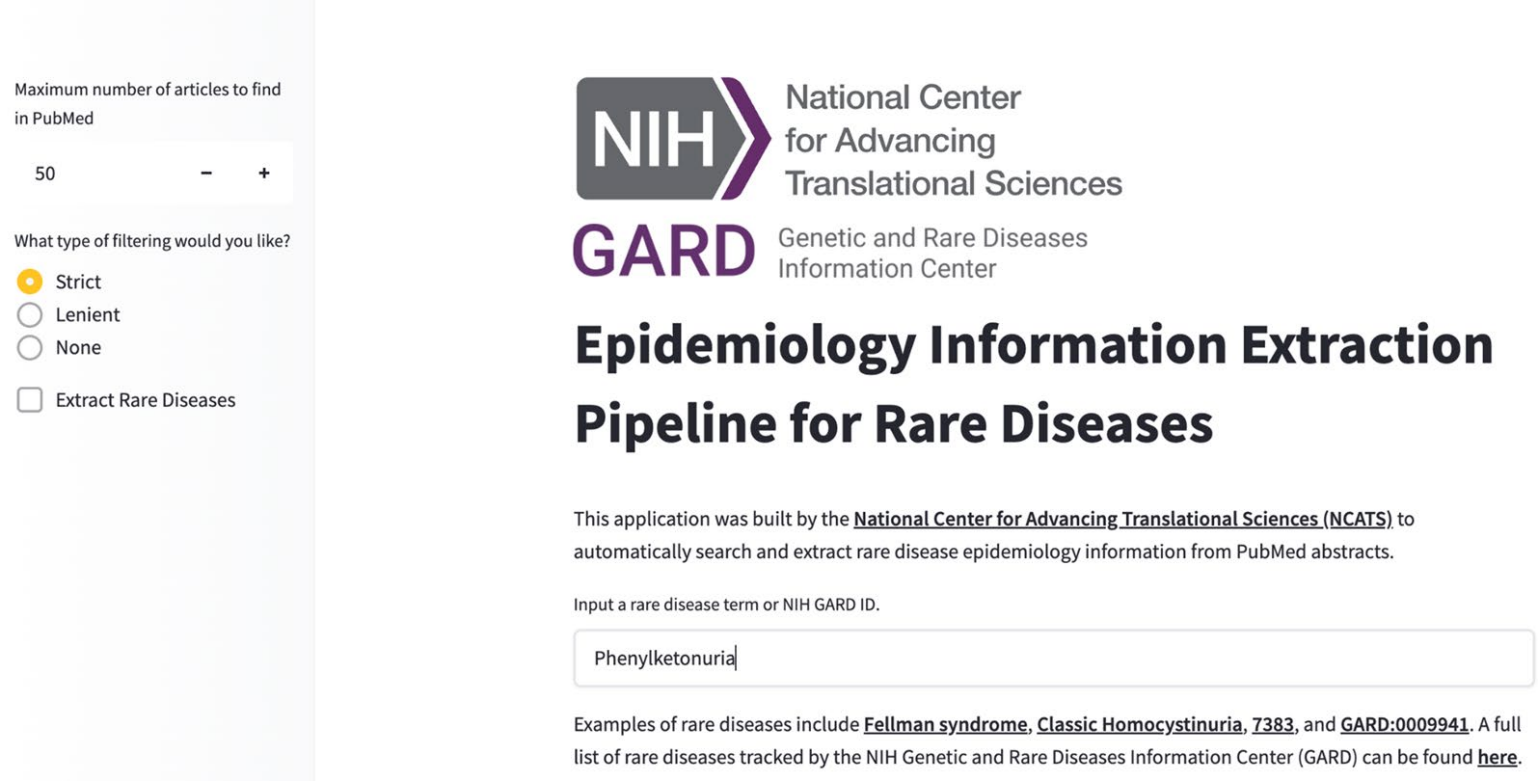
A screenshot of EpiPipeline4RD User Interface

To enable the full capabilities of an automated rare disease EI identification and extraction paradigm, we integrated the aforementioned components into a pipeline named EpiPipeline4RD, which is publicly accessible via a user interface developed on Hugging Face Spaces and API developed with FastAPI: https://rdip2.ncats.io/epihome/documentation.html. A screenshot of the EpiPipeline4RD user interface is shown in Fig. 3.

EpiPipeline4RD takes GARD ID(s) or rare disease name(s) as input(s), and it retrieves all synonyms of the input disease(s) from the NGKG. The PubMed search component then automatically invokes the NCBI and the EBI APIs to gather the input disease(s) relevant PubMed abstracts. During the PubMed searching process, the abstract matching filter can be specified to further filter false positive articles for EI extraction through the UI, i.e., STRICT, excluding articles without mentions of input disease names or synonyms as a whole in the abstracts; LENIENT, excluding articles without mentions of individual tokens composed of input disease names or synonyms in the abstracts; and NO, no filter applied. After that, ES_Predict is initiated to identify ES from the retrieved disease relevant articles, from which the BioBERT model is applied to extract the EI. The extracted EI along with the source PMIDs and their abstracts are shown on the UI in table format and Sankey plot.

## Results

### Dataset Preparation

The EBI RESTful API [57] returned 7,699 unique abstracts for 470 diseases. ES_Predict classified 620 abstracts of the 7,699 abstracts as ES. Notably, 32.8% of the 500 rare diseases had no associated ES. We manually reviewed the 620 abstracts and excluded 11 abstracts which were neither related to rare diseases nor ES. The remaining 609 abstracts were split randomly: fifty abstracts in the test set, 113 abstracts in the hold-out validation set, and 446 abstracts in the training set. After initial pre-processing and labeling the dataset in IOB2 format (Methods 1C) [51, 52], there were 163,060 tokens with labels, of which 7,223 tokens (4.43% of the entire set) were in one of seven entity classes.

**Fig. 4:**
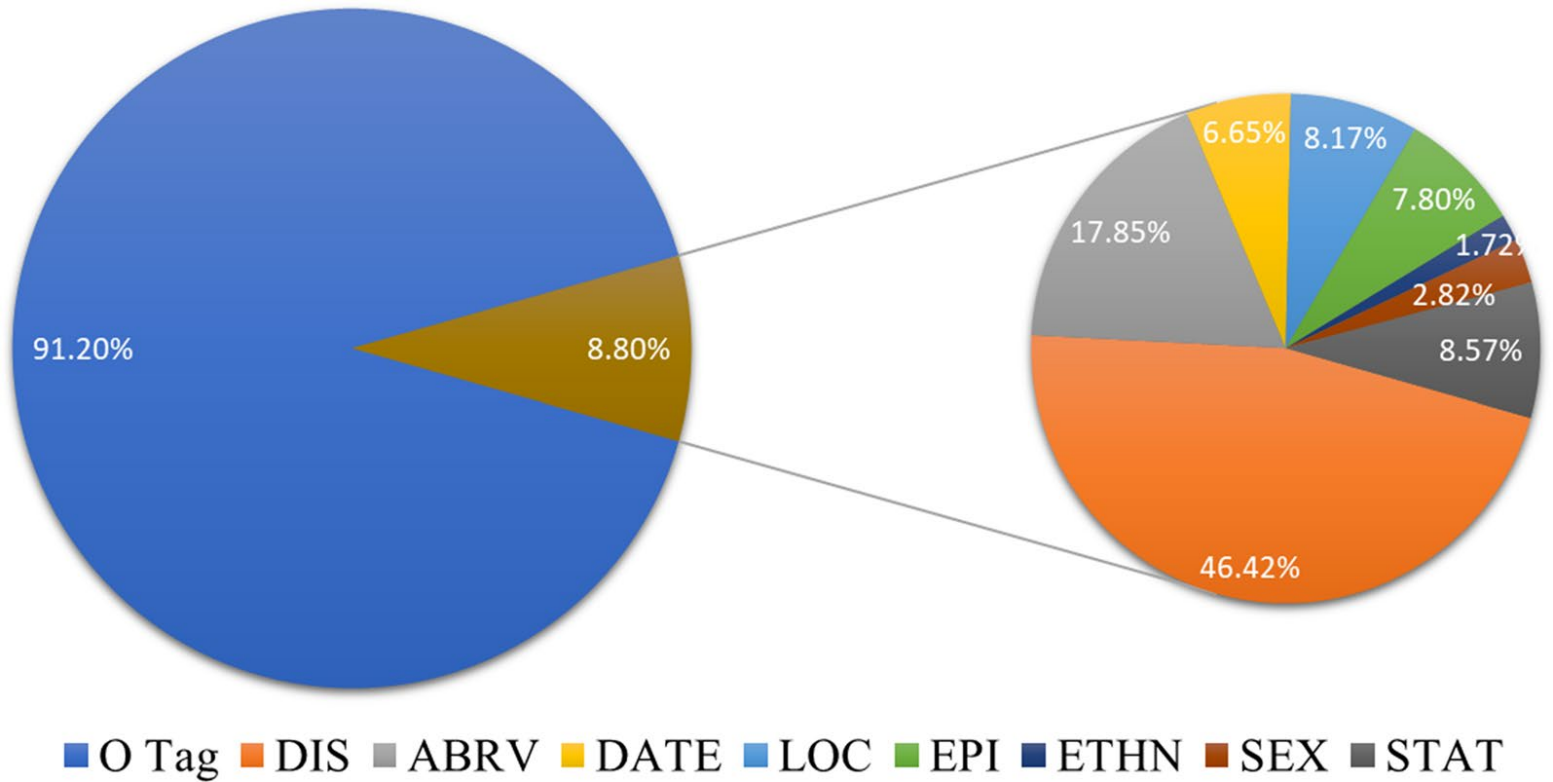
Composition of the entire rare disease epidemiology dataset for named entity recognition (NER)

Thereafter, SMEs manually reviewed and validated the labels generated for seven classes of entities and manually labeled the ABRV entity class. Descriptions of the entity classes are included in Methods 1C. For the first round of validation, 1,693 labels were marked with uncertainty and 441 attached notes explained their uncertainty or the rationale behind the changes made to the labels. For the second round of validation, 1,273 labels were marked with uncertainty and 300 notes added. In the third round, 937 labels were marked as uncertain and attached 339 notes with questions regarding labeling. We further compared those annotations labeled as ETHN to the Ethnicity Ontology [81, 82] and SNOMED Ethnic Group [83] gathered from NCBO BioPortal, and found 74.3% of them overlapped with those two ontologies. Notably “African Americans”, “Brazilian”, “Nepalese”, “Yupik”, and “Roma”, were annotated as ETHN in our dataset, but not found in either of the aforementioned Ethnicity Ontologies. Figure 4 and Table 2 show the composition of the labels in the validated dataset.

**Table 2:** Number of labels in the rare disease epidemiology dataset for NER

Labels	Counts (% of Labels on Tokens)		
	Train set	Validation set	Test set
DIS	5051 (48.96%)	1019 (42.46%)	432 (33.44%)
ABRV	1808 (17.53%)	421(17.54%)	272 (21.05%)
DATE	660 (6.40%)	175 (7.29%)	96 (7.43%)
LOC	764 (7.41%)	262 (10.92%)	118 (9.13%)
EPI	747 (7.24%)	230 (9.58%)	116 (8.98%)
ETHN	192 (1.86%)	33 (1.38%)	16 (1.24%)
SEX	282 (2.73%)	77 (3.21%)	36 (2.79%)
STAT	812 (7.87%)	183 (7.63%)	206 (15.94%)
Sum of all labels (% of Total Labels)	10,316 (9.02% = 10,316/114,425)	2,400 (7.79%)	1,292 (9.29%)
Total (including O tag)	114,425	30,807	13,909

### Model development and fine-tuning

After adapting BioBERT large cased v1.1 for NER, we validated our selection of this pre-trained model by assessing the performance of several existing pre-trained BERT models for NER at the token-level and entity-level. The performance of these models fine-tuned on the dataset is presented in Table 3. BioBERT large v1.1 attained the highest entity-level precision, recall, and F1 scores on predicting locations (LOC), epi types (EPI), epi rates (STAT), dates (DATE), and biological sex (SEX) (Additional file 1), so we chose it as the final pretrained model.

**Table 3:** Comparison metrics of biomedically related pre-trained BERT-based models

Model	Overall token-level precision	Overall entity-level precision
BioBERT large cased v1.1	**0.834**	0.720
BioBERT base cased v1.2	0.825	0.698
PubMedBERT base	0.824	0.713
PubMedBERT + PMC base	0.824	0.715
BlueBERT base	0.829	**0.722**
BlueBERT large	0.818	0.690

The highest scores in each category are bolded. The second highest are underlined

### Dataset fine-tuning

The F1 comparison results to fine turn BioBERT large cased v1.1 on three dataset variants and the standard dataset are shown in Table 4. The “Dataset without DIS and ABRV” with the highest F1 score, was selected as the final dataset for model development.

**Table 4:** Comparison results on Dataset Fine-tuning Variants

Dataset	Entity-level F1
Standard Dataset (with 8 entity classes)	0.755
Dataset with ABRV and DIS merged (with 7 entity classes)	0.750
Dataset without ABRV and DIS (with 6 entity classes)	0.836
Dataset without ABRV, DIS, ETHN (with 5 entity classes)	0.819

### Model optimization

Fine-tuning the BioBERT model achieved the best entity-level results on the holdout validation set after training for 4 epochs (AdamW learning rate = 3e−8 and epsilon = 1e−6 with a linear learning rate scheduler, weight decay = 0.01, warm up ratio = 0.06, gradient checkpointing = True, gradient accumulation = False, 16-bit mixed precision training = False) with a batch size of 16 sentences at a time. The model re-trained with the same hyperparameters and evaluated on the validation set with a batch size of 8 sentences had a loss of 0.0368 and an overall token-level accuracy of 0.992. Precision, recall, and F1 scores are presented at the entity-level and token-level overall and for each entity in Table 5. Clearly, the entity classes with lower degree of variation, are associated with better performance, such as EPI, DATE and SEX. However, because the STAT entity class has various representations in the literature, it increases the complexity/difficulty of recognizing those numbers in the text, and results in comparable low performance. It is worthy to note that the performance of STAT on token-level is better than entity-level, because it is more challenging to recognize the entity of STAT (e.g., “1 in 25 000”) instead of individual tokens (e.g., “1” and “25 000”).

**Table 5:** The performance of BioBERT large cased v1.1 on the Validation Set

Evaluation level	Entity class	Precision	Recall	F1
Entity-level	Overall	0.824	0.851	0.837
	EPI	0.905	0.953	0.929
	STAT	0.688	0.559	0.617
	LOC	0.765	0.761	0.763
	DATE	0.857	0.889	0.873
	SEX	0.949	0.987	0.968
	ETHN	0.571	0.848	0.683
Token-level	Overall	0.921	0.865	0.892
	EPI	0.962	0.962	0.962
	STAT	0.906	0.729	0.808
	LOC	0.936	0.801	0.863
	DATE	0.962	0.989	0.975
	SEX	0.963	0.987	0.975
	ETHN	0.559	0.868	0.680

### Model testing

The model was tested on the “Dataset without ABRV and DIS” test set. The results are presented at the entity-level and token-level in Table 6. The overall accuracy of the model was 0.988.

**Table 6:** The performance of BioBERT large cased v1.1 on the test set

Evaluation level	Entity	Precision	Recall	F1
Entity-level	Overall	0.813	0.821	0.817
	EPI	0.938	0.958	0.948
	STAT	0.522	0.444	0.48
	LOC	0.746	0.770	0.758
	DATE	1.0	1.0	1.0
	SEX	0.923	1.0	0.960
	ETHN	0.65	0.813	0.722
Token-level	Overall	0.932	0.831	0.878
	EPI	0.959	0.967	0.963
	STAT	0.900	0.689	0.780
	LOC	0.959	0.843	0.897
	DATE	1.0	1.0	1.0
	SEX	0.923	1.0	0.960
	ETHN	0.696	0.941	0.800

### Model evaluation by comparing epidemiology data from Orphanet

To qualitatively evaluate the performance of our model for EI extraction, we compared our extracted EI with EI presented in Orphanet [80], and presented five comparisons in Table 7. More comparisons can be found in Additional file 1. For the first three examples shown in Table 7, EI extracted by our model significantly overlaps with EI from Orphanet, although STAT from Orphanet is further normalized in standard range classes and EPI is interpreted (e.g., prevalence at birth or point prevalence vs. prevalence) from the text, which are beyond the scope of this study. The last two examples, Fibrodysplasia ossificans progressiva and Wegener granulomatosis, output two epidemiology statistics without enough context to disambiguate between the prior estimate of prevalence rate and the prevalence rate presented in the study.

**Table 7:** Examples of extracted EI compared with Orphanet data

Diseases	EI labels	BioBERT EI extractor	Orphanet epidemiologic data
Rett syndrome (GARD:0005696), a neurodevelopmental disorder that is characterized by developmental delay and regression, abnormal respiration, absent speech, and inconsolable screaming, crying, panic-like attacks, and gnashing of teeth [84]	Key Phrase from Abstract	“Five patients with definite RS were identified in a population of 203,801 children (98,932 girls) 0–18 years of age yielding a prevalence rate of RS of 1 in 40,760 in North Dakota children.” [85]	
	LOC	North Dakota	United States
	EPI	Prevalence rate(s)	Prevalence at birth
	STAT	1 in 40,760, which can be normalized to 2.45 per 100,000	1–9/100,000
Eosinophilic esophagitis (GARD:0009142), characterized by nausea, vomiting, and pain from inflammation in the esophagus caused by eosinophil invasion [86]	Key Phrase from Abstract	“…series of EE have also been reported in Japan. … The prevalence of EE was calculated to be 17.1/100,000.” [87]	
	LOC	Japan	Japan
	EPI	Prevalence	Point prevalence
	STAT	17.1/100,000	17.1 per 100,000
Smith-Magenis syndrome (GARD:0008197), is “associated with psychomotor delay, a particular behavioural pattern and congenital anomalies.”[88]	Key Phrase from Abstract	“Smith-Magenis syndrome (SMS) is rare (prevalence 1 in 25 000)” [88]	
	LOC	Worldwide	Worldwide
	EPI	Prevalence	Point prevalence
	STAT	1 in 25 000, which is normalized to 4 per 100,000	4 per 100,000
Fibrodysplasia ossificans progressiva (GARD:0006445), an autosomal dominant disorder characterized by a single mutation that leads to the painful ossification of skeletal muscle, tendons, and ligaments after trauma [89]	Key Phrase from Abstract	“Previous studies found that the FOP prevalence was about one in every two million lives. The aim of this study is to estimate the FOP prevalence in France” “89 FOP patients were identified, which results in a prevalence of 1.36 per million inhabitants (CI95% = [1.10; 1.68]).”[90]	
	LOC	France	France
	EPI	Prevalence	Point prevalence
	STAT	1.36 per million inhabitants, which can be normalized to 0.136 per 100,000, and about one in every two million lives	0.136 per 100,000
Granulomatosis with polyangiitis (GARD:0007880), also called Wegener granulomatosis, is an autoimmune disease characterized by sinus and joint pain, respiratory infections, and skin lesions caused by inflammation of blood vessels [91]	Key Phrase from Abstract	“annual incidence/ million population increased from 5.2 (95% confidence interval [95% CI] 2.7–9.0) during 1984–1988 to 12.0 (95% CI 8.0–17.3) during 1994–1998. The point prevalence/million increased from 30.4 (95% CI 16.6–51.0) to 95.1 (95% CI 69.1–129.0).”[92]	
	LOC	Northern Norway	Norway
	EPI	Incidence, incidence rate, annual incidence, prevalence, prevalence rates, point prevalence	Point prevalence
	STAT	Million population, million, 30. 4, to 95. 1	9.51 per 100,000

## Case studies

To demonstrate the capability and performance of our EI extraction pipeline, we performed three case studies via our developed user interface. Our pipeline takes an input of a rare disease term or GARD ID as well as several parameters including the maximum number of abstracts returned from PubMed, type of abstracts filtering, and outputs extracted EI.

**Classic homocystinuria** (GARD:0006667) is an autosomal recessive metabolic disorder caused by mutations in genes necessary for amino acid processing that leads to abnormalities in the ocular, skeletal, and central nervous system if left untreated [93]. Our pipeline searched for 500 studies using all GARD name and synonym term: “homocystinuria due to cystathionine beta-synthase deficiency”, “cystathionine beta-synthase deficiency”, “homocystinuria due to cbs deficiency”, “classic homocystinuria”, and “cbs deficiency”; gathered 105 PubMed IDs; identified 3 ES among them and extracted EI from the abstracts. With our tool, it is easy to overview the incidence of classic homocystinuria across different countries during different time frames. As shown in Table 8, Kuwait has much higher incidence rate than the Czech Republic even with a shorter study time frame. It is worthy to note relation extraction [94–98] is beyond the scope of this study, thus the relationship among those entity classes to the reported ES was not captured. For instance, in the second entry, “Qatar” is extracted as a LOC entity, however it was actually a geographical location which the authors used to compare the incidence rate at Kuwait, rather than the location where the ES was conducted. We also observed that the two ES identified from this case study do not overlap with those listed in Orphanet for classic homocystinuria, since none of the PubMed articles included in the Orphanet were retrieved from PubMed APIs.

**Table 8:** EI Extraction of classic homocystinuria, a subtype of homocystinuria [99]

PubMed Article Title	Newborn population screening for classic homocystinuria by determination of total homocysteine from Guthrie cards [100]	Early diagnosis of classic homocystinuria in Kuwait through newborn screening: a 6-year experience [101]	Vascular presentation of cystathionine beta-synthase deficiency in adulthood [102]
Epi Prob	0.997	0.986	0.862
EPI	Incidence	Incidence	Incidence
STAT	1:1800	1:50,000	1:311,000
LOC	Qatar	Kuwait, Gulf country, Qatar, global	The, Czech Republic
DATE	None	October 2014, between January 2015 and December 2020	Between 1980 and 2009
SEX	None	None	None
ETHN	Qatari	Arabian, Qatari	Czech

**GRACILE syndrome** (GARD:0000001) is a deadly metabolic disease often afflicting infants with iron overload, lactic acid in the bloodstream, amino acids in the urine, and bile stoppage in the liver which leads to growth retardation and early death [103]. Our pipeline identified one ES from a search for 500 PubMed results (search terms: “'growth restriction-aminoaciduria-cholestasis-iron overload-lactic acidosis-early death syndrome”, “growth retardation, aminoaciduria, cholestasis, iron overload, lactic acidosis and early death”, “growth delay-aminoaciduria-cholestasis-iron overload-lactic acidosis-early death syndrome”, “finnish lactic acidosis with hepatic hemosiderosis”, “finnish lethal neonatal metabolic syndrome”, “gracile syndrome”, “fellman syndrome”,and “fellman disease”) and extracted the EI (Table 9). Similar to the first case, we observed that the article [104] referenced for EI extraction by Orphanet is different from the one[105] we retrieved in this case, although both of them are associated with the geographical location of Finland. In addition, no EI for GRACILE syndrome was mentioned in the abstract [105] from Orphanet.

**Table 9:** EI extraction of GRACILE syndrome

Title	The GRACILE syndrome, a neonatal lethal metabolic disorder with iron overload [105]
Epi Prob	0.998
EPI	Incidence
STAT	Least, 1/47,000
LOC	Finland
DATE	None
SEX	None
ETHN	Finnish

**Phenylketonuria** (GARD:0007383) is an autosomal recessive metabolic disorder characterized by an inability to convert excess phenylalanine to tyrosine that is treatable, but can lead to early mental retardation, aggression, and persistent worry if not identified or left untreated. Our pipeline identified three ES from the first 50 returned PubMed results (Search terms: “phenylalanine hydroxylase deficiency”, “oligophrenia phenylpyruvica”, “phenylketonuria”, and “folling disease”) and extracted the EI from the abstracts (Table 10). As mentioned in the first case study, relations among the entity classes were no captured by our pipeline, so correspondences among them are missing, for instance, in the first article with PMID: 34082800, “0.64 per 10, 000 births” (STAT) is the “birth prevalence” (EPI) rate at the “global” (LOC) level, while “0.03 per 10,000 births” (STAT) and “1.18 per 10, 000 births” is the range of “birth prevalence” (EPI) in “the middle east/north africa” (LOC). Similarly, the second article with PMID: 35023679 has “6.002 in 100,000 newborns” (STAT) as the “prevalence” rate (EPI) for “global” (LOC) region and “1 in 4698” (STAT) as the “prevalence” (EPI) in “iran” (LOC).

**Table 10:** EI Extraction of Phenylketonuria

Title	Birth prevalence of phenylalanine hydroxylase deficiency: a systematic literature review and meta-analysis [106]	Frequency of PAH Mutations Among Classic Phenylketon Urea Patients in Mazandaran and Golestan Provinces, North of Iran [107]	Epidemiology of Phenylketonuria Disease in Jordan: Medical and Nutritional Challenges [108]
Epi Prob	0.998	0.997	0.907
EPI	Birth prevalence, birth prevalence, prevalence, birth prevalence estimates, prevalence estimates, birth prevalences	Prevalence	Prevalence
STAT	0.64, per 10,000 births, 0.03, per 10,000 births, 1.18, per 10,000 births, per 10,000 births	6.002 in 100,000 newborns, 1 in 4,698, %, %	1/5263
LOC	global, Southeast Asia, the Middle East/North Africa	of Iran, global, Iran, Mazandaran, north	Jordan, Amman
DATE	None	None	between 2008 and 2021
SEX	None	None	None
ETHN	None	Iranian	Jordanians

## Discussion

Epidemiologic studies provide valuable information to patient groups, researchers, and policy makers. However extraction and curation of epidemiologic information continues to rely primarily on labor-intensive human processes. This study was designed with the intention of augmenting and improving the current paradigm, in order to fulfill the UN resolution for collection and analysis of disaggregated data on rare disease persons [27]. Here we presented a newly generated EI corpus and a rare disease based EI extraction pipeline named EpiPipeline4RD consisting of ES_Predict, a long short-term memory recurrent neural network for ES identification, and a bidirectional, transformer-based, deep learning model for EI extraction. Furthermore, we developed a user interface to freely access our pipeline. Identifying and extracting EI from rare disease literature at scale is an exceptionally difficult challenge, but this work represents the state of the art in an effort to reduce the human effort required to curate and analyze EI from rare disease literature. Ultimately, we hope this effort can begin to shift the paradigm towards an integrative approach to rare disease support that mitigates the efficiency and sustainability challenges for rare disease epidemiology posed by Halley et al. [109]

We created the first-in-class dataset for rare disease epidemiology NER in the IOB2 format which not only effectively supports EI extraction, but also offers more opportunities to improve predictive performance [110], support multi-label sentence classification, and be an NLP benchmark dataset for future studies. In our corpus, we labeled eight entity classes relevant to EI based on consultation with our SMEs and prior literature [64, 111]. Although disease concepts (DIS) and disease abbreviations (ABRV) were not included in our BioBERT model, as disease extraction is beyond the scope of this study, they were captured and included in our corpus due to three reasons for our future enhancement. First, it allows related diseases or possible comorbidities which might be co-factors considered in the ES to be captured. Second, it helps to disambiguate complicated relationships of multiple diseases and their associated EI in text. For instance in the abstract, “Krabbe disease was the most common (one in 39 000) followed by Gaucher disease (one in 47 000), metachromatic leukodystrophy and Salla disease” [112]. In this case, identifying those disease terms will be the first step for relation extraction [113] linking the diseases to their EI, ultimately to construct knowledge graphs for the epidemiology of each rare disease. Third, given the fact that a disease term is normally mentioned at the beginning of the abstract, and then referenced again with its abbreviation in the subsequent sentences where the EI is stated [114]. In this case, coreference resolution [115, 116] is required to unambiguously link the disease abbreviation with its corresponding disease.

As shown in Tables 6, 7, 8, 9, our BioBERT model illustrated high quantitative and qualitative performance of extracting EI from PubMed articles. Understanding the model’s performance on individual entity classes reveals important information which highlights possible routes for future improvement. The model performed well on identifying entities in the EPI and SEX classes which have little variation in their representations. EPI reaches an F1 of 0.948 at the entity-level and an F1 of 0.962 the token-level. Though there were fewer SEX entities labeled in the training set (2.73%) and relatively low diversity of presentation of SEX entities in the training, validation, and test sets, it would result in good performance on common biological sex phrases. However, it may not be able to identify more complex phrases of biological sex such as “XYY” and “intersex”. Thus, we propose extending our current corpus by adding more SEX and other annotations from diverse literature to improve the performance of our model.

**Fig. 5:**
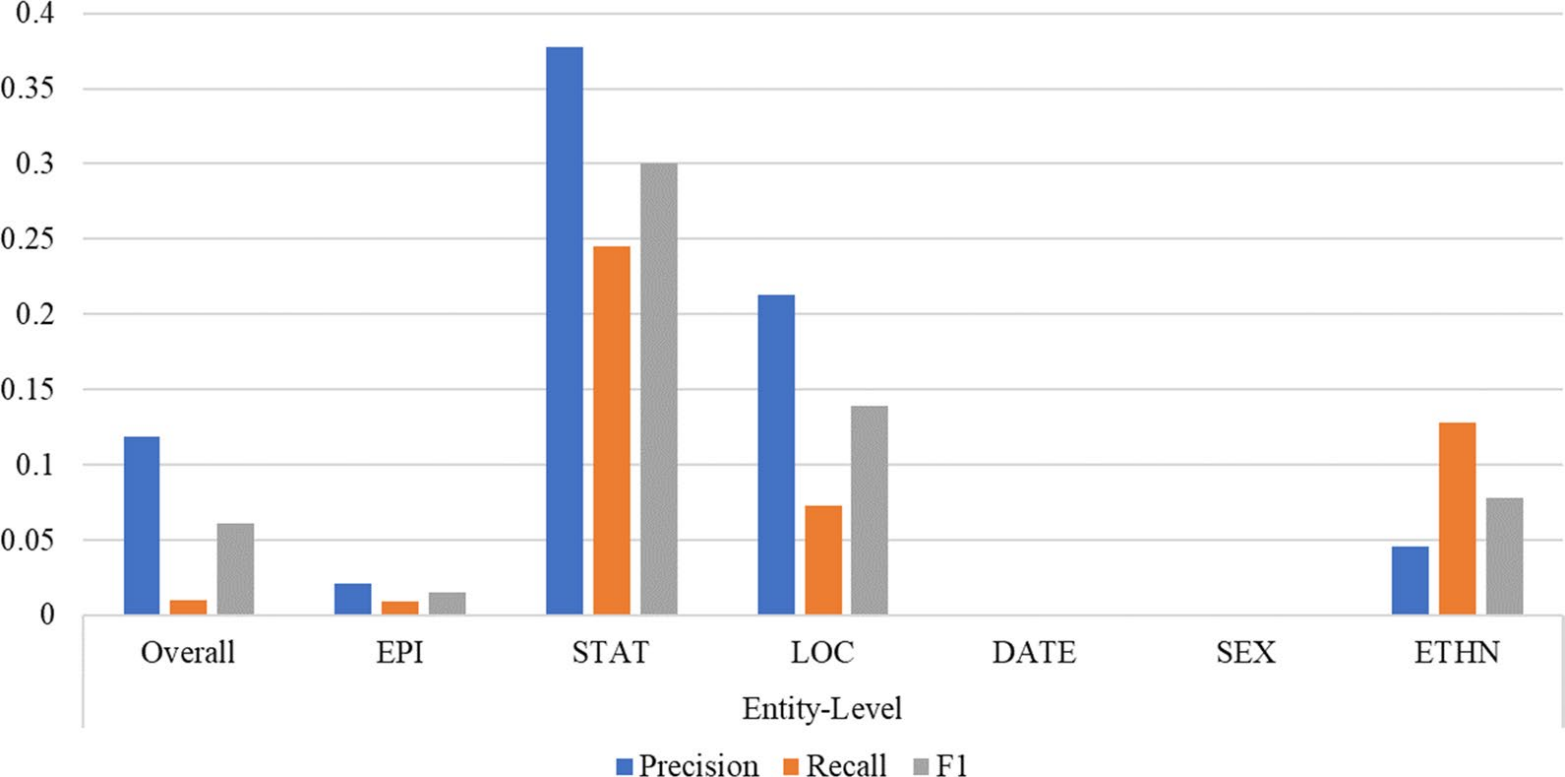
Absolute difference between token-level and entity-level test results

The entity classes of LOC, ETHN, and STAT showed high disparities between token-level and entity-level results (Fig. 5), due to the lower degrees of variation and complexity of token representations rather than entity representations. Though the training dataset contained more LOC labels than EPI, DATE, and SEX, the model achieved lower performance on LOC than each of those classes with token-level and entity-level F1 scores of 0.897 and 0.758 respectively on the test set. This is likely attributed to the wide diversity of location information as well as a large number of multi-word entities. Due to a sparsity of ETHN training data (1.86%) and ETHN entities often overlap with LOC entities, the model sometimes misclassified LOC and ETHN. For instance, in the first abstract of the Classic Homocystinuria case study, the token “Ara” is common to the location “Arabian Gulf” and the word “Arab” which was labeled as ETHN in the training set, so the model misclassified the whole word “Arabian” as an ETHN rather than as a LOC. Obviously contextual information is critical for the model to discriminate between LOC and ETHN, so it requires more training data. More training data from rare disease literature or bootstrapped from existing sources such as the CoNLL +  + dataset [117, 118] may provide avenues to algorithmically improve noisy and distantly supervised learning as well as increase the robustness of the model in the future study.

As BERT-based models use word-pieces to encode digits, the limited performance of the model on STAT entities at the entity-level may be further explained through an understanding of its numeracy. BERT-based models have been shown to have some limitations on numeracy, thus the BERT-based model relies much more heavily on contextual information and the attention mechanism rather than sub-word embeddings to differentiate the significance between different numbers [119]. Without numeracy, floating point numbers which dominate the STAT entity class in cases such as “0. 50 / 10, 000 girls.” [65], are not determinically represented. Utilizing Deterministic, Independent-of-Corpus Embeddings [120], NumBERT’s scientific notation [121], or a combination in NumGPT [122] may strengthen the model’s ability to differentiate numbers on the basis of numeration and magnitude.

Rare disease names and synonyms represent an extremely diverse, unique, lengthy nomenclature. On the other extreme, the ABRV entity class often represents a complex disease concept in a few characters which may overlap with other concepts when uncased (i.e. “ChILD” [123] being represented as “child”, MS [124], and CS [125]). The model’s initial performance on the DIS and ABRV entity classes, which were labeled in the dataset but not utilized in the final model’s training, could potentially be attributed to BioBERT’s limited WordPiece embeddings for rare disease and disease abbreviation concepts.

Qualitatively, the comparison with Orphanet in Table 6 indicated that our model can achieve comparable results to Orphanet on some extractions. Given the primary focus of GARD is to manage rare disease following the US definition [1], our model would effectively assist the GARD curation effort to systematically capture U.S.-based EI with specificity at the state level. This is exemplified by the comparison of “Rett Syndrome” as our model extracted “1 in 40, 760” as STAT and “North Dakota” as LOC compared to Orphanet curating “1-9/100,000” as the prevalence rate and “United States” as the location because they report epidemiologic rates as range classes when they do not have enough data to give an accurate value for the larger regions and normalize their location data to country, continent, or worldwide [4].

The performed case studies demonstrated efficiency, validity, and thoroughness of our integrated pipeline to extract relevant EI for rare diseases with high precision. Interestingly, we found that a few PubMed articles referenced by Orphanet for the diseases presented in the three case studies were not identified in our pipeline due to the EBI and NCBI APIs, whereas we identified articles which were not present in Orphanet either. Furthermore, we noticed that the results returned from these two APIs differed significantly and, based on a small sample of searches, quantified the difference using the Jaccard index [126]. For instance, the highest similarity between the lists of PubMed articles returned from these two APIs is about 0.52 by searching ‘Morphea’, and the lowest similarity score is about 0.025 by searching ‘Santos Mateus Leal syndrome’. Thus, we opted to combine the EBI and NCBI APIs to increase the number of PubMed articles for further analysis. In addition, we implemented two searching strategies to further exclude false positives: 1) given the high performance of the LSTM RNN based ES_Predict, only disease terms and ES_Predict were applied to invoke the EBI and NCBI APIs for rare disease epidemiology article retrieval and identification from PubMed. 2) STRICT filtering with an in-text rare disease identification algorithm (Additional file 2) allows the pipeline to be robust when the EBI and NCBI APIs return articles unrelated to the queried search term.

With the aforementioned limitations, several extensions are proposed for the next step. We will focus on decreasing the latency of the pipeline; increasing the variety of EPI such as R0, prevalence rate ratio, pooled frequency, etc.; implementing superior machine learning algorithms such as Knowledge-supervised Deep Learning [52]; utilizing larger language models trained in the biomedical domain; and building upon yet-to-be invented artificial intelligence architectures. Furthermore, in order to capture EI beyond epidemiologic studies, our model framework with improved numeracy could be applied to extract information and aggregated case or family counts from case reports. In a similar manner, due to the generalizability of pre-trained deep bidirectional transformers, our approach could also be repurposed for multi-type token classifiers for clinical trials, natural history studies, or literature types in domains beyond rare diseases.

## Supplementary Information

**Additional file 1.** Supplementary Methods 1 describes two algorithms used in the training and deployment of the epidemiology information extraction model and pipeline. The first algorithm describes in pseudocode how data used for training the BioBERT model was prelabeled in a weakly-supervised fashion. The second algorithm describes a method used to identify rare disease terms in abstracts.

**Additional file 2.** Supplementary Methods 2 describes the guidelines used by GARD researchers to manually improve and validate the pre-labeled training dataset.

**Additional file 3.** Supplementary Data includes four datasheets. The first is “Pretrained Model Validation” which shows the performance of each pretrained model on the validated dataset at entity-level and token-level both overall with microaveraging and broken down by entity class. The second is “Dataset Annotation Counts 1" which contains the raw numbers of each tag in each dataset and other summary statistics about the manually validated dataset. The third is “Dataset Annotation Counts 2” which contains a pie chart that summarizes Dataset Annotation Counts 1. The fourth is “Filtered Orphanet Comparison” which contains the results of the model’s comparison to Orphanet, filtered to only include results where our disease identification algorithm identified 1 or less GARD IDs and our model identified at least one STAT in text. The left side contains Orphanet’s extraction. The right contains our extraction. The middle is the source both are drawing from.

## Data Availability

The full comparison, code, and other supplemental data, including test predictions, is found on GitHub. The final fine-tuned model and dataset are available for download and use on Hugging Face with the *transformers *[67] and *datasets* [127] Python packages respectively, links to all are found here: https://rdip2.ncats.io/epihome/documentation.html.
