# Correction: Precision information extraction for rare disease epidemiology at scale

**DOI:** 10.1186/s12967-023-04127-1

**Published:** 2023-04-29

**Authors:** William Z. Kariampuzha, Gioconda Alyea, Sue Qu, Jaleal Sanjak, Ewy Mathé, Eric Sid, Haley Chatelaine, Arjun Yadaw, Yanji Xu, Qian Zhu

**Affiliations:** 1 Division of Rare Diseases Research Innovation, National Center for Advancing Translational Sciences (NCATS), National Institutes of Health (NIH), Bethesda, MD USA; 2 Division of Pre-Clinical Innovation, National Center for Advancing Translational Sciences (NCATS), National Institutes of Health (NIH), 9800 Medical Center Drive, Rockville, MD 20850 USA

**Correction: Journal of Translational Medicine (2023) 21:157** 10.1186/s12967-023-04011-y

Following publication of the original article [1], we have been notified that one of the authors’ names was misspelled in the Acknowledgement section—Alexey Zakharov. It should be as follows:

**Acknowledgements**

The authors thank Alexey Zakharov, Jorge Neyra and Dac-Trung Nguyen for their thoughtful feedback and technical implementation as well as Richard Pacheco, Eduardo Luiggi, Carlin Biyoo, Ke Wang and Hugo Hernandez for infrastructure support.
