# Supplementary material for: Precision information extraction for rare disease epidemiology at scale
Source: J Transl Med. 2023 Feb 28;21:157. doi: 10.1186/s12967-023-04011-y (PMC9972634; doi:10.1186/s12967-023-04011-y)
Supplement: Supplementary file 2 — Additional file 2. Supplementary Methods 2 describes the guidelines used by GARD researchers to manually improve and validate the pre-labeled training dataset. [file 12967_2023_4011_MOESM2_ESM.pdf]

Precision Information Extraction for Rare Disease Epidemiology at Scale: A Deep Learning Approach
Supplementary Methods 1

William Z. Kariampuzha, B.S.,
Gioconda Alyea, M.D., Chunxu Qu, Ph.D.,
Haley Chatelaine, Ph.D., Jaleal Sanjak, Ph.D., Arjun Yadaw, Ph.D.,
Ewy Mathé, Ph.D., Eric Sid, M.D., Yanji Xu, Ph.D.,
Qian Zhu, Ph.D.

February 2022

1 Pre-Labeling Algorithm

Input : List of sentences from a Rare Disease Epidemiology Abstract Corpus
Output: List of tokens and IOB2 labels

Algorithm runs in $O(n)$ time.

1.1 Define Sets of Terms to Label:

The set of epidemiological (epi) types we wish to label are constructed from four sets of terms gathered from a corpus analysis:

1. epi roots = {incidence(s),prevalence(s),occurrence(s), prevalent}
2. epi conditional-roots = {affect(s), occurs, frequency, frequencies}
3. epi pre-root-modifiers = {annual, overall, estimated, weighted, nationwide, pooled, average, cumulative, annualized, age-adjusted, sex-adjusted, associated, population-based, calculated, combined, corrected, familial, race/ethnicity-specific, race-specific, birth, community-based, point, total, age-specific, ethnicity-specific}
4. epi post-root-modifiers = {estimate(s), rate(s)}

Set of epi types = all phrases of the forms

- "pre-root-modifier::root::post-root-modifier"
- "pre-root-modifier::root"
- "root::post-root-modifier"
- "root"

Set of conditional epi types = all phrases of the forms

- "pre-root-modifier::conditional-root::post-root-modifier"
- "pre-root-modifier::conditional-root"
- "conditional-root::post-root-modifier"

Note: Conditional phrases are only labeled when an epidemiologic rate is in the same sentence.

Set of Ethnicities, Nationalities, & Races = unprocessed and stemmed terms gathered from Wikipedia

Set of Rare Diseases = disease names and synonyms from GARD Knowledge graph

Set of Biological Sexes = {male(s), female(s), girl(s), boy(s), man, men, woman, women, intersex, XYY, XXXY, XXXXY, klinefelter syndrome, klinefelter}

Note: Turner syndrome is not included because it is already a rare disease

Set of Dates = all dates from January 1, 1900 – December 31, 2021 in the forms (day month year), (month day year), (month day , year), (month day, year), (month year), (year)

Set of Epi Rate Modifiers = {between, around, approximately, about, <, >, roughly, relatively, over, under, than}

Set of Range Terms = {-, -, –, –, —, —, –, and, to, until}

Note: These terms indicate that two entities may be connected e.g. June 2021 – February 2022 or 1/100,000 - 3/100,000 live births

1.2 Function used in the algorithm

```
Function combineEntities(tokens : list, labels : list) is
    for each (token, label) in lists of tokens and labels do
        if token is in set of range words then
            if token is '<' OR '>' then
                label ← STAT
            end
            if token is 'than' then
                label ← STAT
                label_before ← STAT
            end
            if (token is 'birth(s)' OR 'LB(s)') AND previous label is STAT then
                label ← STAT
            end
            if (label before token = DATE AND label after token = DATE then
                label ← DATE
            end
            if label before token = STAT AND label after token = STAT then
                label ← STAT
            end
        end
    end
end
```

1.3 Pre-Labeling Algorithm

```
for each sentence in corpus do
    list of tokens ← spaCyTokenize(sentence) ;
    list of labels ← list of 'O' with length = list of tokens
    for each token in list of tokens do
        entity ← getspaCyEntity(token)
        if entity is a geopolitical entity or a location then
            label ← LOC ← spaCyLabel(token)
        end
        if token is 'global' OR 'worldwide' then
            label ← LOC
        end
        if first character of token is a digit AND ('/' OR ':' is in the token) AND 'ratio' not
          in phrase then
            label ← STAT
        end
        if entity is a percent AND phrase <1% AND nearby tokens do not indicate that the
          text is in a 'confidence interval' then
            label nearby phrase as STAT
        end
        if token is 'one' OR '1' then
            label following phrase as STAT
        end
        if token is 'per' AND (a nearby token is a digit OR a nearby entity is a cardinal
          number, ordinal number, quantity, or money) then
            label nearby phrase as STAT
        end
        if token is 'unknown' AND an epi_type is in the sentence then
            label ← STAT
        end
        end_token ← token at end of sentence
        for each phrase from token to end_token do
            if phrase is in set of rare diseases then
                label all tokens in phrase as DIS
            end
            else if phrase is in set of epi types then
                label all tokens in phrase as EPI
            end
            else if phrase is in set of epi conditional types AND an epi rate is in the sentence
              then
                label all tokens in phrase as EPI
            end
            else if phrase is in set of biological sexes then
                label all tokens in phrase as SEX
            end
            else if phrase is in set of dates then
                label all tokens in phrase as DATE
            end
            if raw phrase or stemmed phrase is in set of ethnicities/nationalities/races then
                label all tokens in phrase as ETHN
            end
            end_token ← token before end_token in sentence
        end
    end
    list of tokens and labels ← combineEntities(list of tokens, list of labels)
end
```

2 Disease Identification Algorithm

2.1 Variables used in disease identification

Rare Disease Dictionary = keys are disease names and synonyms, values are the respective GARD ID numbers from GARD Knowledge graph

maxLength = the number of words in the longest disease name or synonym in the Rare Disease Dictionary

2.2 Disease Identification

Input : Sentence from a Rare Disease Epidemiology Abstract Corpus
Output: List of disease names/synonyms, List of GARD IDs

```
list of tokens ← NLTK_Tokenizer(sentence)
i ← 0
diseaseList ← emptyList
GARDidList ← emptyList
while i < the length of the list of tokens do
    compareLength ← returnMinimumValue(length of list of tokens from token i to end,
      maxLength)
    while compareLength > 0 do
        phrase ← joinTokens(token at position i, token at position compareLength)
        if phrase is in GARD Dictionary keys then
            add phrase to diseaseList
            add corresponding GARD ID from GARD Dictionary to GARDidList
        end
        compareLength ← compareLength -1
    end
end
```
