# Supplementary material for: Precision information extraction for rare disease epidemiology at scale
Source: J Transl Med. 2023 Feb 28;21:157. doi: 10.1186/s12967-023-04011-y (PMC9972634; doi:10.1186/s12967-023-04011-y)
Supplement: Supplementary file 3 — Additional file 3. Supplementary Data includes four datasheets. The first is “Pretrained Model Validation” which shows the performance of each pretrained model on the validated dataset at entity-level and token-level both overall with microaveraging and broken down by entity class. The second is “Dataset Annotation Counts 1" which contains the raw numbers of each tag in each dataset and other summary statistics about the manually validated dataset. The third is “Dataset Annotation Counts 2” which contains a pie chart that summarizes Dataset Annotation Counts 1. The fourth is “Filtered Orphanet Comparison” which contains the results of the model’s comparison to Orphanet, filtered to only include results where our disease identification algorithm identified 1 or less GARD IDs and our model identified at least one STAT in text. The left side contains Orphanet’s extraction. The right contains our extraction. The middle is the source both are drawing from. [file 12967_2023_4011_MOESM3_ESM.docx]

**Precision Information Extraction for Rare Disease Epidemiology at Scale**

**Supplementary Methods 2:**

**Gold-Standard Epidemiology Dataset** **Guidelines for Labeling**

**William Z. Kariampuzha, B.S.^1^, Gioconda Alyea, M.D., M.S.^2^, Chunxu Qu, Ph.D.^1^, Haley Chatelaine, Ph.D.^3^, Jaleal Sanjak, Ph.D.^3^, Arjun Yadaw, Ph.D.^3^, Ewy Mathé, Ph.D.^3^, Eric Sid, M.D.^1^, Yanji Xu, Ph.D.^1^, Qian Zhu, Ph.D.*^3^**

**^1^Office of Rare Diseases Research (ORDR), National Center for Advancing Translational Sciences (NCATS), National Institutes of Health (NIH), Rockville, MD; ^2^ICF International Inc, Rockville, MD; ^3^Division of Pre-Clinical Innovation, National Center for Advancing Translational Sciences (NCATS), National Institutes of Health (NIH), Rockville, MD**

# **Introduction**

The goal of this project is to use deep learning (DL) to extract epidemiological (epi) information from rare disease epidemiological abstracts. To do so, we must provide the DL model with a dataset of high-quality examples to learn from.

The first step of our process to create this dataset was gathering a corpus of rare disease epidemiology abstracts. The second step was utilizing weakly supervised machine learning techniques and natural language processing to initially label the epidemiological information in the dataset. We have completed these steps. The third step is to manually validate those labels to create a gold-standard (highest quality) dataset with labeled epidemiological information for this task (named entity recognition, NER). Below we describe guidelines for this manual validation step.

The final dataset should have 8 classes (or entity types) of epidemiological information labeled: ***disease*** names & synonyms ***(DIS)***; ***disease*** ***abbreviations (ABRV)***; ***locations*** and geopolitical entities ***(LOC)***; ***dates*** ***(DATE)*** (e.g. inner quotes: “Data were collected from ‘January 2002 to September 2019’”); ***ethnicities/nationalities/races (ETHN)*** (e.g. “Italian”, “Ashkenazi Jew”, “Marshallese”); ***biological sex (SEX)***; ***epi types (EPI)*** (e.g. “incidence”, “prevalence”, “frequency”); and ***epi rates (STAT)*** (“approximately 1 in 40,000 live births”). Anything that is not one of these categories should be labeled as **O**. Each token (i.e. a word, punctuation mark, abbreviation, etc.) in each abstract will be assigned a label.

# **Descriptions of Entity Classes**

**EPI label.** Based on de la Paz et al.,[1] we identified and labeled epidemiologic types such as “prevalence(s)”, “incidence(s)”, “affects”, and “occurrence(s)” as EPI. Guided by our GARD subject matter experts (SMEs) (GA and ES) as well as the precedent set by Orphanet,[2] epidemiologic types such as mortality rate, case fatality rate, R_0_, prevalence rate ratio, pooled frequency, positive predictive value, and recurrence rate were deemed to be out-of-scope for this study. To capture contextual information surrounding the aforementioned epidemiologic types, we implemented a concordance analysis with AntConc,[3] which allowed us to label their associated inflections and modifiers (e.g. “nationwide”, “estimated”, and “annual”) along with the epidemiologic types as EPI.

**STAT label.** We labeled phrases with epidemiologic rates, such as “14.1 cases per 1,000 live births” and “<1/500,000”, as STAT by using the spaCy 3[4, 5] *en_core_web_lg* NER model. Meanwhile we iteratively generated heuristic rules to label STAT. To exemplify a few rules, numbers with “/” or phrases with the word “per” likely indicated rates, percentages that begin with “0.*%” were likely to be a percent-based rate, if the word “unknown” and an EPI label were in the same sentence then “unknown” was likely to be an epidemiologic rate. A complete collection of those heuristic rules can be found in the Supplemental Methods 1. Given suggestions from our SMEs, we excluded epidemiologic rates within confidence intervals, which often split single entities across a span, posing an additional challenge for the BioBERT model.

**DIS and ABRV labels.** We labeled rare disease terms as DIS using exact string match to the GARD disease names or their synonyms retrieved from the NGKG,[6] but opted to label non-rare diseases during the next step of manual validation to minimize false positives. Disease abbreviations were labeled as a different entity class of ABRV to support coreference resolution in a future study. However, it is challenging to accurately label rare disease abbreviations from the text because abbreviations are frequently presented as disease synonyms (some of them were initially labeled as DIS while labeling the disease entity). Thus, we opted to label all disease abbreviations ABRV during the manual validation step.

**ETHN label.** It is worthy to note that prevalence rate can vary across different subgroups with different ethnicities,[7, 8] races,[9, 10] and nationalities.[11-14] Thus, we opted to include ETHN as an entity class. To reduce the complexity of labeling the related concepts of ethnicities, races, and nationalities, we conflated them into a single entity class of ETHN. Given the variety of representations of these concepts in PubMed abstracts, we constructed an ethnicity dictionary (accessible via <https://github.com/ncats/epi4GARD/blob/master/epi_extract_datasets/ethnicities.json>) by scraping words and phrases that indicated ethnicity, nationality, or race from Wikipedia to label tokens in our dataset as ETHN.

**DATE, SEX, and LOC labels.** As certain rare diseases such as malaria[10] and Rett syndrome[15] have differential epidemiology rates based on biological sex, curating currently unknown sexual dichotomies between disorders such as 17 alpha hydroxylase deficiency[16] or Bardet-Biedl syndrome 10,[17] may offer critical information to health systems and researchers. Thus, we labeled biological sex as SEX according to the heuristic that certain phrases such as “boys”, “females”, “XYY”, and “Klinefelter syndrome” were likely to indicate the sex chromosomes of study participants. For dates regarding when the ES was conducted or when the data was gathered, we programmatically recognized and labeled date related phrases as DATE based on the Document Automation Language date formats 4, J, and their simplified variants.[18] For locations where the ES took place, we utilized spaCy NER to directly label tokens as LOC. We also adopted the labeling strategy from Orphanet to label the words “global” and “worldwide” as LOC.[2]

# **Labeling Format**

We utilize the IOB2 format (Inside-Outsize-Beginning Format 2)[19, 20] to label the tokens. The first token (beginning) of a phrase is labeled with ***B-{label}*** and subsequent parts of the phrase are listed with ***I-{label}*** and tokens that are not in a phrase are labeled with ***O***, where ***{label}*** is any of the eight entity types such as ***LOC*** or ***STAT***. The end of an entity phrase can be inferred by the model when ***I-{label}*** is followed by ***O***. Detailed rules for each label discussed in the following sections.

## **Diseases**

Label all disease names and synonyms (including non-rare diseases) even they are not pre-labeled as ***{B or I}-DIS*** (Table 1).

Do not mark symptoms, features, or phenotypes of diseases as **diseases**, even they were discussed like diseases. Do not mark **abbreviations** as **diseases**. If the phrase has a unique identifier or code as a disease (e.g. ICD, GARD, UMLS) then mark it.

**Table 1:** Example of labeling of a disease name or synonym

| Wegener's | B-DIS |
| --- | --- |
| Granulomatosis | I-DIS |

Include disease modifiers/adjectives such as *chronic* and *acute* as a part of the disease phrase. Table 2 also shows that non-common adjectives such as *juvenile onset* can offer important information to GARD curators so should be labeled in the disease phrase:

**Table 2:** Example of labeling a disease with modifiers/adjectives^[21]^

| juvenile | onset | diabetes | mellitus | ( | DM | ) |
| --- | --- | --- | --- | --- | --- | --- |
| B-DIS | I-DIS | I-DIS | I-DIS | O | B-ABRV | O |

Label *pathogenic infections* as **diseases**, however, do not label the *organism* as **diseases.** For example, label *COVID-19* as **B-ABRV**; *coronavirus disease 2019* as **B-DIS, I-DIS, I-DIS**; *SARS-CoV-2* as **O**.

**Disease** **Abbreviations**

Some abstracts only used disease abbreviation without using disease name. Additionally, it is important to distinguish between disease abbreviations and disease names/synonyms for a separate natural language processing task of coreference resolution. Therefore, disease abbreviation has a separate label from disease. Label abbreviations of diseases as ABRV (Table 2, 3). Follow all other constraints from the disease entity class, including not marking symptoms nor pathogens. For instance, label *Zika virus* as **O, O**; *ZIKV* as **O**; but *ZIKV infection* as **B-ABRV, I-ABRV.**

**Table 3:** Example of labeling a disease abbreviation

| **Token** | Most | amyotrophic | lateral | sclerosis | ( | ALS | ) |
| --- | --- | --- | --- | --- | --- | --- | --- |
| **Label** | O | B-DIS | I-DIS | I-DIS | O | B-ABRV | O |

Occasionally abbreviations are infixed in the disease term*.* Abbreviation infixes should be labeled as in Table 4. Mark **C** (for *come back*) in the **Unsure** column to indicate that there is an infix there. Mark “Y” for any label or phrase where the labeling is uncertain. Suppose these guidelines did not make the distinction between pathogens and diseases clear and it was unclear whether to mark “herpes simplex virus” as a disease. In this instance, the appropriate procedure would be to mark “Y” (for *yes* in the unsure column).

**Table 4:** Example of utilizing the unsure column^[22]^

| **Token** | **Label** | **Unsure** |
| --- | --- | --- |
| Background | O |  |
| National | O |  |
| neonatal | O |  |
| surveillance | O |  |
| for | O |  |
| herpes | B-DIS |  |
| simplex | I-DIS |  |
| virus | I-DIS | Y |
| ( | O |  |
| HSV | B-ABRV | C |
| ) | O |  |
| disease | I-DIS |  |

Table 5 is an example of not marking symptoms or features of diseases even when discussed like diseases (notice, hypertension is not labeled).

**Table 5:** Differentiating between symptoms of diseases and diseases.^[23]^

| **Token** | **Label** |
| --- | --- |
| The | O |
| prevalence | B-EPI |
| of | O |
| comorbidities | O |
| was | O |
| as | O |
| follows | O |
| : | O |
| hypertension | O |
| 8 | O |
| % | O |
| , | O |
| diabetes | B-DIS |
| mellitus | I-DIS |
| 8 | O |
| % | O |

**Locations**

Label all locations according to the IOB2 format e.g. **{B or I}-LOC**. If US population is mentioned or indicated, label *US* as **B-LOC**. If it is indicated to be *worldwide* or *global*, label it as **B-LOC**. If specific geographical locations are referenced, label them (*Italy, Norway*), region (*Eastern Europe, Central Asia*), or continent (*Europe, North America*) as well (Table 6).

**Table 6:** Example of a correctly labeled location

| North | B-LOC |
| --- | --- |
| - | I-LOC |
| Central | I-LOC |
| Africa | I-LOC |

Label as much of a phrase as a location as possible (towards ground truth). Table 7 compares a fully labeled phrase to an incompletely labeled phrase.

**Table 7:** Correct vs incorrect labeling of locations^[24]^

| **Token** | **Correct Labeling** | **Incorrect Labeling** |
| --- | --- | --- |
| 11 | B-LOC | O |
| coastal | I-LOC | O |
| villages | I-LOC | O |
| from | I-LOC | O |
| the | I-LOC | O |
| northern | I-LOC | O |
| Bering | I-LOC | B-LOC |
| Sea | I-LOC | I-LOC |
| to | I-LOC | O |
| the | I-LOC | B-LOC |
| Beaufort | I-LOC | I-LOC |
| Sea | I-LOC | I-LOC |
| . | O | O |

## **Biological Sex**

A non-exhaustive list of examples to be labeled as **{B or I}-SEX.** Label according to the heuristic that some tokens are likely to indicate the biological sex of the study participants.

| - male(s) | - boy(s) | - woman | - intersex |
| --- | --- | --- | --- |
| - female(s) | - man | - women | - XXXY |
| - girl(s) | - men | - XYY | - XXXXY |

**Race, Ethnicity, & Nationality**

If available, label any ethnicities in text as **{B or I}-ETHN**. Here is a non-exhaustive list of examples (more listed at end):

| - American Indian - Alaska Native - Americans - Asian - Black or African American - Hispanic or Latino - Puerto Rican - Native Hawaiian | - Pacific Islander - Marshallese - White - Italian - Ashkenazi Jewish - French Canadian - Mediterranean |
| --- | --- |

## **Dates**

Label all dates and date ranges as **{B or I}-DATE**. For example, *From 2009 through 2018* should be labeled as [**O, B-DATE, I-DATE, I-DATE**]. Additional examples are listed in the EPI rate section and in Tables 15 and 16.

## **Epi Types**

For this this type of entity, each phrase is broken into three parts “pre-root_modifier::root::post-root_modifier”, where modifiers may or may not be present. Label these as **{B or I}-EPI**.

Label this list of roots in all cases, except when the EPI rate or disease is not valid (i.e. when it refers to a symptom of a disease):

- Incidence(s)
- prevalence(s)
- occurrence(s)

Label this list of roots if the root refers to an EPI rate:

- Affect
- Occurs
- Affects
- Frequency
- Frequencies

These are pre-root modifiers that we found from a corpus concordance analysis[3] that should be labeled:

| - Annualized - Age-adjusted - Sex-adjusted - Annual - Annualized - Associated - Population-based - Calculated - Combined - Corrected - Cumulative - Estimated - Familial | - race/ethnicity-specific - Race-specific - Birth - Average - Birth - Community-based - Overall - Point - Total - Age-specific - Ethnicity-specific - Overall |
| --- | --- |

These are post-root modifiers that were identified from the corpus analysis:

- estimate(s)
- rate(s)

Thus, some possible epi type phrases would be *annualized incidence* (labeled: **B-EPI, I-EPI**), *occurrence* (labeled: **B-EPI**), or Table 8:

**Table 8:** Example of labeling epidemiology type phrases

| **Token** | point | prevalence | estimates |
| --- | --- | --- | --- |
| **Label** | B-EPI | I-EPI | I-EPI |

If other useful modifiers are identified, they should be labeled and the document should be updated accordingly with a comment stating the updates.

Do not label these phrases or anything with these roots:

| - Prevalence rate ratio - Incidence risk ratio - Standardized incidence ratio(s) - Pooled incidence(s) - Prevalence ratio | - Pooled prevalence(s) - Pooled occurrence(s) - Mortality rate - Case fatality rate - Incidence ratio |
| --- | --- |

## **Epi Rates**

These are the most complex entities in terms of variety of presentation. Some examples: *estimated at 1 in 200,000*, *affects approximately 1 in 100,000 Americans* (where *Americans* would be labeled as **B-ETHN**) and *affects 1 in 7500 to 1 in 10,000 people* which would be labeled as [**B-EPI, B-STAT, I-STAT, I-STAT, I-STAT, I-STAT, I-STAT, I-STAT, I-STAT**]. They can be presented as simply as Table 9 or as complex as Table 10.

**Table 9:** Example of epidemiological rates

| approximately | 5 | cases | per | million | individuals | annually |
| --- | --- | --- | --- | --- | --- | --- |
| B-STAT | I-STAT | I-STAT | I-STAT | I-STAT | I-STAT | I-STAT |

As seen in the Table 9, include all modifiers before and after the core epidemiological rate phrase such as *approximately* and *individuals annually*. Because it is not possible to provide an exhaustive list as it was done with the epi types, use the guidelines and judgement when considering the semantics of the phrases.

| **Table 10:** A complex presentation of epi rates^[25]^   \| **Token** \| **Label** \| **Unsure** \| \| --- \| --- \| --- \| \| The \| O \|  \| \| annual \| B-EPI \|  \| \| incidence/ \| I-EPI \|  \| \| million \| I-EPI \| Y \| \| population \| I-EPI \| Y \| \| increased \| O \|  \| \| from \| O \|  \| \| 5.2 \| B-STAT \|  \| \| ( \| O \|  \| \| 95 \| O \|  \| \| % \| O \|  \| \| confidence \| O \|  \| \| interval \| O \|  \| \| [ \| O \|  \| \| 95 \| O \|  \| \| % \| O \|  \| \| CI \| O \|  \| \| ] \| O \|  \| \| 2.7- \| O \|  \| \| 9.0 \| O \|  \| \| ) \| O \|  \| \| during \| O \|  \| \| 1984-1988 \| B-DATE \|  \| \| to \| I-STAT \| Y \| \| 12.0 \| I-STAT \| C \| \| ( \| O \|  \| \| 95 \| O \|  \| \| % \| O \|  \| \| CI \| O \|  \| \| 8.0 \| O \|  \| \| - \| O \|  \| \| 17.3 \| O \|  \| \| ) \| O \|  \| \| during \| O \|  \| \| 1994-1998 \| B-DATE \|  \| \| . \| O \|  \| | In this instance, it is necessary to individually analyze what should be included. As an example in Table 10, */ million population* was included as a part of the epidemiology type (**EPI**) because if it was not labeled then it would not make sense. If it was labeled as **B-STAT** (like most */million* phrases would be) then it would not be connected with the rest of the epidemiology rate phrases and the extraction would look like epi type: [*incidence]* and epi_rate: *[/ million population, 5.2, 12.0*], which would not be easy to read. The way it is currently labeled would “teach” the model to extract: **epi type**: {*incidence/ million population*} and **epi_rate**: [*5.2, 12.0*], which is still not easy to read, but preferred to the previous example. |
| --- | --- | --- | --- | --- | --- | --- | --- | --- | --- | --- | --- | --- | --- | --- | --- | --- | --- | --- | --- | --- | --- | --- | --- | --- | --- | --- | --- | --- | --- | --- | --- | --- | --- | --- | --- | --- | --- | --- | --- | --- | --- | --- | --- | --- | --- | --- | --- | --- | --- | --- | --- | --- | --- | --- | --- | --- | --- | --- | --- | --- | --- | --- | --- | --- | --- | --- | --- | --- | --- | --- | --- | --- | --- | --- | --- | --- | --- | --- | --- | --- | --- | --- | --- | --- | --- | --- | --- | --- | --- | --- | --- | --- | --- | --- | --- | --- | --- | --- | --- | --- | --- | --- | --- | --- | --- | --- | --- | --- | --- | --- | --- | --- |

**Some other important guidelines:**

- Rates can be presented as percentages too!
- Rates should be marked even if they are the rates in a particular population such as just “*women*” or just “*Ashkenazi Jews*”.
- Do not mark p-values or confidence intervals (Table 10)
- Do not mark the rates of case fatality or mortality (or any other epi type that is disallowed)
- Do not mark a rate that refers to a symptom of a disease or group of diseases (this is difficult to tell and will require you checking to see if the disease was labeled or not). Only mark rates that refer to diseases, even if it is a non-rare disease.
- If the text indicates that the epi rate is “*unknown*”, mark that as **B-STAT**

**Table 11:** Example of labeling an epidemiology rate of a non-rare disease^[26]^

| and | O |  |  |
| --- | --- | --- | --- |
| Cryptosporidium | O |  |  |
| spp | O |  |  |
| . | O |  |  |
|  |  |  |  |
| infection | O |  |  |
| among | O |  |  |
| Iranian | B-ETHN |  |  |
| general | O |  |  |
| population | O |  |  |
| were | O |  |  |
| calculated | O |  |  |
| 1.3 | B-STAT | Y | LABELED BECAUSE IT IS A VALID STAT OF A DISEASE, NOT NECESSARILY A RARE DISEASE? |
| % | I-STAT | Y |  |

- For multiple rates strung together, label the longest contiguous phrase possible (Table 12).

**Table 12:** Labeling multiple rates with a common decedent^[27]^

| **Tokens** | **Labels** |
| --- | --- |
| 0.79 | B-STAT |
| , | I-STAT |
| 1.12 | I-STAT |
| , | I-STAT |
| 1.22 | I-STAT |
| and | I-STAT |
| 6.04 | I-STAT |
| per | I-STAT |
| 100000 | I-STAT |
| newborns | I-STAT |
| in | O |
| Asia | B-LOC |
| - | I-LOC |
| Pacific | I-LOC |
| , | O |
| Europe | B-LOC |
| , | O |
| North | B-LOC |
| America | I-LOC |
| and | O |

**Table 13:** Labeling a range of epidemiology rates^[28]^

Note that *ranges from* is included

| **Token** | **Label** |
| --- | --- |
| The | O |
| prevalence | B-EPI |
| ranges | B-STAT |
| from | I-STAT |
| 1.3 | I-STAT |
| % | I-STAT |
| to | I-STAT |
| 8.8 | I-STAT |
| % | I-STAT |
| of | I-STAT |
| all | I-STAT |
| cholecystectomies | I-STAT |
| and | O |
| varies | O |
| from | O |
| country | O |
| to | O |
| country | O |

# **Split rates**: EPI rates that are split by confidence interval (or other) infixes should be treated as one phrase, continue labeling the words after the infixes as **I-STAT**. The infixes should be marked with **C** in the **Unsure** column so that we can find them easily later.

**Table 14:** Labeling split rates and marking infixes^[29]^

| **Token** | **Label** | **Unsure** |
| --- | --- | --- |
| The | O |  |
| overall | O |  |
| annualized | B-EPI |  |
| incidence | I-EPI |  |
| of | O |  |
| granuloma | B-DIS |  |
| annulare | I-DIS |  |
| was | O |  |
| 0.04 | B-STAT |  |
| % | I-STAT |  |
| , | O |  |
| or | O |  |
| 37.9 | B-STAT |  |
| ( | O |  |
| 95 | O | C |
| % | O |  |
| CI | O |  |
| , | O |  |
| 36.9 | O |  |
| - | O |  |
| 38.9 | O |  |
| ) | O |  |
| per | I-STAT |  |
| 100000 | I-STAT |  |

#

#

# **Fully Labeled Examples**

| **Table 15:** Fully labeled example #1^[7]^   \| **Token** \| **Label** \| \| --- \| --- \| \| To \| O \| \| characterize \| O \| \| their \| O \| \| Illness \| O \| \| , \| O \| \| we \| O \| \| reviewed \| O \| \| ADH \| O \| \| surveillance \| O \| \| reports \| O \| \| of \| O \| \| HD \| O \| \| among \| O \| \| Marshallese \| B-ETHN \| \| persons \| O \| \| in \| O \| \| Arkansas \| B-LOC \| \| treated \| O \| \| during \| O \| \| 2003-2017 \| O \| \| (n = \| O \| \| 42) \| O \| \| . \| O \| \|  \|  \| \| Hansen's \| B-DIS \| \| Disease \| I-DIS \| \| prevalence \| B-EPI \| \| among \| O \| \| Marshallese \| B-ETHN \| \| in \| O \| \| Arkansas \| B-LOC \| \| ( \| O \| \| 11.7/10000 \| B-STAT \| \| ) \| O \| \| was \| O \| \| greater \| O \| \| than \| O \| \| that \| O \| \| in \| O \| \| the \| O \| \| general \| O \| \| U.S. \| O \| \| population \| O \| \| . \| O \| | **Table 16:** Fully labeled example #2^[30]^   \| **Token** \| **Label** \| \| --- \| --- \| \| Background \| O \| \| Reported \| O \| \| birth \| B-EPI \| \| prevalences \| I-EPI \| \| of \| O \| \| congenital \| O \| \| limb \| O \| \| defects \| O \| \| ( \| O \| \| CLD \| O \| \| ) \| O \| \| vary \| O \| \| between \| O \| \| countries \| O \| \| : \| O \| \| from \| O \| \| 13/10,000 \| O \| \| in \| O \| \| Finland \| B-LOC \| \| for \| O \| \| the \| O \| \| period \| O \| \| 1964 \| B-DATE \| \| - \| I-DATE \| \| 1977 \| I-DATE \| \| to \| O \| \| 30.4/10,000 \| O \| \| births \| O \| \| in \| O \| \| Scotland \| B-LOC \| \| from \| O \| \| 1964 \| B-DATE \| \| - \| I-DATE \| \| 1968 \| I-DATE \| \| . \| O \| \|  \|  \| \| Conclusions \| O \| \| The \| O \| \| birth \| B-EPI \| \| prevalence \| I-EPI \| \| of \| O \| \| CLD \| O \| \| in \| O \| \| the \| O \| \| northern \| B-LOC \| \| Netherlands \| I-LOC \| \| was \| O \| \| 21.1/10,000 \| O \| \| births \| O \| \| . \| O \| |
| --- | --- | --- | --- | --- | --- | --- | --- | --- | --- | --- | --- | --- | --- | --- | --- | --- | --- | --- | --- | --- | --- | --- | --- | --- | --- | --- | --- | --- | --- | --- | --- | --- | --- | --- | --- | --- | --- | --- | --- | --- | --- | --- | --- | --- | --- | --- | --- | --- | --- | --- | --- | --- | --- | --- | --- | --- | --- | --- | --- | --- | --- | --- | --- | --- | --- | --- | --- | --- | --- | --- | --- | --- | --- | --- | --- | --- | --- | --- | --- | --- | --- | --- | --- | --- | --- | --- | --- | --- | --- | --- | --- | --- | --- | --- | --- | --- | --- | --- | --- | --- | --- | --- | --- | --- | --- | --- | --- | --- | --- | --- | --- | --- | --- | --- | --- | --- | --- | --- | --- | --- | --- | --- | --- | --- | --- | --- | --- | --- | --- | --- | --- | --- | --- | --- | --- | --- | --- | --- | --- | --- | --- | --- | --- | --- | --- | --- | --- | --- | --- | --- | --- | --- | --- | --- | --- | --- | --- | --- | --- | --- | --- | --- | --- | --- | --- | --- | --- | --- | --- | --- | --- | --- | --- | --- | --- | --- | --- | --- | --- | --- | --- | --- | --- | --- | --- | --- | --- | --- | --- | --- | --- | --- | --- |

**References**

1. de la Paz MP, Villaverde-Hueso A, Alonso V, Janos S, Zurriaga O, Pollan M, Abaitua-Borda I: Rare diseases epidemiology research**.** In *Advances in Experimental Medicine and Biology.* *Volume* 686. Edited by Posada de la Paz M, Groft S: Springer Science+Business Media B.V.; 2010: 17-39

2. Orphanet: Procedural document on Epidemiology of rare disease in Orphanet (Prevalence, incidence and number of published

cases or families)**.** Orphanet; 2019.

3. Anthony L: AntConc**.** 3.4.3 edition. Tokyo, Japan: Waseda University; 2014.

4. Honnibal M, & Montani, I: spaCy 2: Natural language understanding with Bloom embeddings, convolutional neural networks and incremental parsing**.** 2017.

5. Honnibal M, Johnson M: An Improved Non-monotonic Transition System for Dependency Parsing**.** In *2015 Conference on Empirical Methods in Natural Language Processing; Lisbon, Portugal*. Association for Computational Linguistics; Sept 2015: 1373–1378.

6. Zhu Q, Nguyen DT, Grishagin I, Southall N, Sid E, Pariser A. An integrative knowledge graph for rare diseases, derived from the Genetic and Rare Diseases Information Center (GARD)**.** J Biomed Semantics. 2020; 11(1)**:**13.

7. Labuda SM, Williams SH, Mukasa LN, McGhee L. Hansen's Disease and Complications among Marshallese Persons Residing in Northwest Arkansas, 2003-2017**.** Am J Trop Med Hyg. 2020; 103(5)**:**1810-1812.

8. Mehta A. Epidemiology and natural history of Gaucher's disease**.** Eur J Intern Med. 2006; 17 Suppl(S2-5.

9. McGarry ME, Gibb ER, Oates GR, Schechter MS. Left behind: The potential impact of CFTR modulators on racial and ethnic disparities in cystic fibrosis**.** Paediatr Respir Rev. 2021.

10. Hussin N, Lim YA, Goh PP, William T, Jelip J, Mudin RN. Updates on malaria incidence and profile in Malaysia from 2013 to 2017**.** Malar J. 2020; 19(1)**:**55.

11. Ramos JM, Romero D, Belinchon I. Epidemiology of Leprosy in Spain: The Role of the International Migration**.** PLoS Negl Trop Dis. 2016; 10(3)**:**e0004321.

12. Lastoria JC, Abreu MA. Leprosy: review of the epidemiological, clinical, and etiopathogenic aspects - part 1**.** An Bras Dermatol. 2014; 89(2)**:**205-218.

13. Staphylococcal food poisoning**.** National Center for Advancing Translational Sciences; 2021.

14. Lee WC, Sakai T, Lee MJ, Hamakawa M, Lee SM, Lee IM. An epidemiological study of food poisoning in Korea and Japan**.** Int J Food Microbiol. 1996; 29(2-3)**:**141-148.

15. Suzuki H, Hirayama Y, Arima M. [Prevalence of Rett syndrome in Tokyo]**.** No To Hattatsu. 1989; 21(5)**:**430-433.

16. 17-alpha-hydroxylase deficiency**.** National Center for Advancing Translational Sciences; 2021.

17. Bardet-Biedl syndrome 10**.** National Center for Advancing Translational Sciences; 2021.

18. Oracle: Date Format Types**.** 2014.

19. Sang EF, Veenstra J: Representing text chunks**.** In *arXiv preprint*; 1999.

20. Ramshaw LA, Marcus MP: Text chunking using transformation-based learning**.** In *Natural language processing using very large corpora.* Springer; 1999: 157-176

21. Hwang WJ, Lim HH, Kim Y-M, Chang MY, Kil HR, Kim JY, Song WJ, Levy HL, Kim S-Z. Pancreatic involvement in patients with inborn errors of metabolism**.** Orphanet Journal of Rare Diseases. 2021; 16(1)**:**1-9.

22. Berkhout A, Kapoor V, Heney C, Jones CA, Clark JE, Vaska VL, Lai M, Nourse C. Herpes simplex virus infection in infants: 13 year evaluation (2005–2017) of laboratory confirmed cases in Queensland, Australia**.** The Pediatric Infectious Disease Journal. 2021; 40(3)**:**209-214.

23. Al-Sadawi M, Singh K, Capric V, Mohiuddin A, Haddadin M, Nunez A, Shaikh S, Bukharovich I, McFarlane SI. Incidence and Associated Risk Factors of Chemotherapy-Induced Cardiomyopathy in the African American and Afro-Caribbean Populations**.** International journal of clinical research & trials. 2020; 5(

24. Huntington HP, Quakenbush LT, Nelson M. Effects of changing sea ice on marine mammals and subsistence hunters in northern Alaska from traditional knowledge interviews**.** Biology letters. 2016; 12(8)**:**20160198.

25. Koldingsnes W, Nossent H. Epidemiology of Wegener's granulomatosis in northern Norway**.** Arthritis Rheum. 2000; 43(11)**:**2481-2487.

26. Mohebali M, Keshavarz H, Afshar MJA, Hanafi-Bojd AA, Hassanpour G. Spatial Distribution of Common Pathogenic Human Intestinal Protozoa in Iran: A Systematic Review**.** Iranian Journal of Public Health. 2021; 50(1)**:**69.

27. Almási T, Guey LT, Lukacs C, Vokó Z, Zelei T. Systematic literature review and meta-analysis on the epidemiology of methylmalonic acidemia (MMA) with a focus on MMA caused by methylmalonyl-CoA mutase (mut) deficiency**.** Orphanet journal of rare diseases. 2019; 14(1)**:**1-10.

28. Giudicelli X, Rode A, Bancel B, Nguyen A-T, Mabrut J-Y. Xanthogranulomatous cholecystitis: Diagnosis and management**.** Journal of visceral surgery. 2021; 158(4)**:**326-336.

29. Barbieri JS, Rodriguez O, Rosenbach M, Margolis D. Incidence and prevalence of granuloma annulare in the United States**.** JAMA dermatology. 2021; 157(7)**:**824-830.

30. Vasluian E, van der Sluis CK, van Essen AJ, Bergman JE, Dijkstra PU, Reinders-Messelink HA, de Walle HE. Birth prevalence for congenital limb defects in the northern Netherlands: a 30-year population-based study**.** BMC musculoskeletal disorders. 2013; 14(1)**:**1-14.
